# A modular metagenomics analysis system for integrated multi-step data exploration

**DOI:** 10.1101/2023.04.09.536171

**Published:** 2023-04-09

**Authors:** Lauren Mak, Braden Tierney, Cynthia Ronkowski, Michael Toomey, Juan Sebastian Andrade Martinez, Sam Zimmerman, Chenlian Fu, Malika Kopbayeva, Anna Noyvert, Brett Farthing, Shuiquan Tang, Christopher Mason, Iman Hajirasouliha

**Affiliations:** 1 Tri-Institutional Computational Biology & Medicine Program, Weill Cornell Medicine of Cornell University, NY, USA and; 2 Institute for Computational Biomedicine, Weill Cornell Medicine, New York, NY, 10065, USA and; 3 Department of Physiology and Biophysics, Weill Cornell Medicine, New York, NY, 10065, USA and; 4 Department of Clinical Pharmacy, School of Pharmacy, University of Southern California, Los Angeles, CA 90089, USA and; 5 Section on Pathophysiology and Molecular Pharmacology, Joslin Diabetes Center, Boston, MA, USA and; 6 Department of Microbiology, Harvard Medical School, Boston, MA, USA and; 7 Zymo Research, 17062 Murphy Ave, Irvine, CA 92614 and; 8 WorldQuant Initiative for Quantitative Prediction, Weill Cornell Medicine, New York, NY, USA and; 9 The Feil Family Brain and Mind Research Institute, Weill Cornell Medicine, New York, NY, USA and; 10 Englander Institute for Precision Medicine, The Meyer Cancer Center, Weill Cornell Medicine, NY, USA

## Abstract

**Motivation:**

Computational analysis of large-scale metagenomics sequencing datasets has proved to be both incredibly valuable for extracting isolate-level taxonomic and functional insights from complex microbial communities. However, thanks to an ever-expanding ecosystem of metagenomics-specific algorithms and file formats, designing studies, implementing seamless and scalable end-to-end workflows, and exploring the massive amounts of output data have become studies unto themselves. Furthermore, there is little inter-communication between output data of different analytic purposes, such as short-read classification and metagenome assembled genomes (MAG) reconstruction. One-click pipelines have helped to organize these tools into targeted workflows, but they suffer from general compatibility and maintainability issues.

**Results:**

To address the gap in easily extensible yet robustly distributable metagenomics workflows, we have developed a module-based metagenomics analysis system written in Snakemake, a popular workflow management system, along with a standardized module and working directory architecture. Each module can be run independently or conjointly with a series of others to produce the target data format (ex. short-read preprocessing alone, or short-read preprocessing followed by *de novo* assembly), and outputs aggregated summary statistics reports and semi-guided Jupyter notebook-based visualizations, The module system is a bioinformatics-optimzied scaffold designed to be rapidly iterated upon by the research community at large.

**Availability:**

The module template as well as the modules described below can be found at https://github.com/MetaSUB-CAMP.

## 1 Introduction

Metagenomics refers to sequencing-based investigation of all micro-organisms within environmental or host-associated microbial communities. These communities are typically complex mixtures of dozens to hundreds of species. Since many organisms cannot be cultured for targeted sequencing, the taxonomic and functional properties of the community must be recovered from aggregated shotgun metagenomic sequencing data. This wet-to-dry workflow has yielded some incredible insights into the ecology and evolution of samples taken from environments such as (Almeida et al. (2019); Danko et al. (2021); Kadosh et al. (2020); Brito et al. (2019); Sierra et al. (2022)).

The dry component of the metagenomics analysis workflow is responsible for processing sequencing data to extract study-specific taxonomic and/or functional information. Typically, the taxonomic unit of choice for downstream analysis is the metagenome-assembled genome, or MAG, a microbial isolate genome reconstructed from sequencing data. Alternative strategies involve classifying sequencing reads directly, or characterizing the functional content of the entire community through gene annotation of *de novo* assembled contigs (Quince et al. (2017)).

One of the main challenges of metagenomic analysis is the organization and application of multiple computational tools into workflows to extract biologically relevant and interpretable insights from raw sequencing reads. While there is a vast array of open-source tools available, many of them are not accessible online, easy to install, or eventually installable at all (Mangul et al. (2019)). Open-source package management systems such as Conda and containerization systems such as Nextflow and Snakemake have partially addressed these challenges, dependency conflicts and operating incompatibilities are still major problems when multiple tools need to coexist in the same environment (ana (2020); Merkel (2014); Kurtzer (2018)). Of the tools that are easy to install and use, a significantly smaller subset of them have been benchmarked on realistic simulated datasets, making their strengths, weaknesses, and use-cases difficult to determine (Sczyrba et al. (2017); McIntyre et al. (2017); Zhang et al. (2022); Meyer et al. (2018)).

However, all-in-one pipelines come with a consistent set of challenges. Because they wrap so many tools, there are many dependent points of failure at both installation and run time. Pipelines that do not support workload managers such as Slurm and instead rely on containerization are not widely usable on high-performance compute clusters with root restrictions. Many pipelines balance ease of use with parameter complexity by heavily favouring the former and masking the parameter ranges of the encapsulated tools, usually hard-coding them as the default value. All-in-ones tend to be developed and maintained by a single laboratory, and are limited in terms of end-user customization potential. Because all-in-one pipelines are designed to take end-users from input to desired output in one go, the intermediate results are implicitly obscured, preventing the end-user from integrating their domain expertise and allowing manual curation to play a role in data cleaning and analysis. As has been demonstrated, in-depth intermediate data exploration is an integral part in ensuring MAG quality, especially in the reconstruction of circularized and (near-)complete microbial isolate genomes (Chen et al. (2019); Moss et al. (2020)).

An alternative way to conceptualize metagenomics analysis is to design workflows themselves as an interconnected suite of modules. To that end, we have designed a modular metagenomics analysis system comprised of several self-contained workflows and semi-guided visualizations, called modules. Each module is designed to complete a single analytic task (ex. *de novo* assembly), accepting a standardized input format (ex. CSV of paths to FastQ files) generated by antecedent modules (ex. read preprocessing), and generating a standardized output format(s) (ex. CSV of paths to assembled contigs, summary statistics report). While each module wraps a different set of analytic tools, every module shares a common commandline interface, module directory structure, and working directory structure, facilitating intuitive data navigation by the user as well as numerous customization opportunities. By wrapping Snakemake internals using this command-line interface, our modular workflow facilitates the following analytic utilities:

- **‘Set menu’-style computing to ‘a la carte’-style study design**: Switching from one-click pipelines to a modular analysis system allows the user can assemble the exact workflow for the exact study purpose they want by downloading only the modules required to return the target data.
- **Built-in soft pauses at the end of each module**: At the conclusion of each module, or ‘step’ in the workflow, the user can explore several semi-automated visualizations of their analytic results, and apply their own knowledge base to empower downstream analyses, such as adjusting downstream parameters from default values.
- **Modules as benchmarking and comparison meta-tools:** A module can be used as a benchmarking ‘sandbox’ (similar to the Snakemake pipeline by Orjuela et al. (2022)) where new algorithms can be easily added to a workflow and then compared to other algorithms with the same objective.
- **Compressed summary statistics and semi-guided visualizations:** Essential for large-scale dataset analysis, hypothesis generation, and reliability assessments.
- **Module template for future expansions**: Each module is based on a standard directory template. New modules for new analysis purposes can be easily set up from scratch using the cookiecutter command into a within a few hours complete with Conda environments and module-specific parameter and resources files.

## 2 Methods

### 2.1 Module Structure

Each module is a Github repository with the following core components in a standardized directory structure so all of the module directory (Box 2.1.1), working directory, parametrization and input/output architectures are consistent across the system. The Snakefile, utils.py, ext/ directory, parameter.yaml, and resource.yaml are further customized for the module’s specific purpose. The contents of each module as well as the encapsulated tools involved in short-read taxonomic classification and MAG inference and quality control are described in the Supplementary References. Each of the design features of the modular system is described and highlighted in Table 1.

#### 2.1.1 Workflow Language

The modular metagenomics analysis system is a suite of Snakemake-based modules that integrate several custom features on top of Snakemake’s capabilities that wrap state-of-the-art bioinformatic tools for metagenomics analysis. These modules can be used separately for a single targeted purpose or one after another in an end-to-end workflow, mimicking one-click pipelines. The current modules available are designed to facilitate the analysis of short sequencing reads using one of two methods: i) short-read taxonomic classification, and ii) MAG reconstruction and quality-checking. Future modules that address other sequencing technologies (ex. long reads) and other analysis goals (ex. phage hunting) are currently in development.

While Nextflow (Di Tommaso et al. (2017)) and Snakemake (Mölder et al. (2021)) are both popular choices as workflow management systems for computational biology analysis, we chose Snakemake for its general readability and accessibility for researchers who want to start with analytic backbone and customize from there. As with Nextflow, Snakemake has standard pipelining features such as intermediary file handling, rule reexecution in the case of job failure, batch job scheduler (ex. Slurm) integration, cloud computing support, Conda-based package management, and log writing.

#### 2.1.2 Environment Management

We have chosen not to implement the modular system with Docker (Merkel (2014)) or Singularity (Kurtzer (2018)) integration for the reason that many users of high-performance compute clusters (HPCs) lack root access privileges and thus cannot use containerized workflows. Instead, we have added conflict-free recipes that each set up a Conda environment built directly into the module directory so that the same environment can be used for all datasets processed using that same module (ana (2020)).

### 2.2 Results

#### 2.2.1 Modular System Use-cases

The primary strength of a modular system is in its flexibility and extensibility, not only by the developers of the module template and core modules, but by anyone who has a fundamental command line, Python, and Conda development skillset. A few example use-cases are listed below.

1. User who has a short-read dataset and wants to do a first-pass analysis with short-read taxonomic classification: The user downloads two modules- short-read preprocessing to eliminate low-quality and error bases, and short-read taxonomic classification- to generate a merged report of discovered taxa from three classification tools. The user only has to learn one command-line interface as the CLI is universal across all modules.
2. Above user wants to extend their analysis to gene annotation: The user simply has to download and run two more modules-short-read assembly and gene cataloging. There is no extra onboarding time required in terms of learning an interface, and the input to short-read assembly is the same as short-read taxonomic classification- the samples.csv containing the paths to the preprocessed short reads.
3. Above user wants to extend their analysis to MAG reconstruction: The user simply has to download the binning and MAG quality-checking modules and use the output of short-read assembly- the samples.csv with paths to the *de novo* assemblies- as their input. Again, no extra onboarding time for learning new interfaces.
4. User who has developed a new short-read taxonomic classification tool: The user can use the existing short-read taxonomic module as a sandbox, write a custom Snakemake rule to run their tool, write a function to harmonize their output format with the existing merged report, and benchmark their tool for strengths and weaknesses.
5. User wants to specifically study extra-chromosonal elements: Since a module for this purpose does not currently exist, the user can create a new blank module using the Cookiecutter template, populate it with the appropriate Snakemake rules, and customize the rest of the demo configs for their exact purpose. Because of the standardized input-output format, this new extra-chromosonal elements module can be integrated seamlessly with the rest of the module system for reuse by the developer and the wider research community.

#### 2.2.2 Proof-of-Concept

To demonstrate the analytic capabilities of a modular system, we ran the five modules described in the Supplementary Materials on two human gut microbiome samples, each of which were sequenced three times to compare average dataset quality, assembly sizes, and taxonomic classifications across multiple technical replicates. These six sequencing datasets were generated as part of the Microbiome-in-a-Bottle project, where the objective is to overlap the species inferences from sequencing human gut microbiomes using multiple technologies (ex. short-read, long-read, linked-read, Hi-C) to arrive at a single consensus community composition. Paired with the sets of real sequencing data, this community composition will serve as a gold-standard benchmarking resource for metagenomics tool development by the field at large.

From sample 1, we were able to retrieve 7,134 species at the short-read level and 47 species at the MAG level. From sample 2, we were able to retrieve 8,436 species at the short-read level in total and 82 species at the MAG level. We found that the inter-module pauses were essential to understanding the analysis process and the biological and technical properties of our sequencing datasets.

#### 2.2.3 Short-Read Preprocessing

**Fig. 1.**
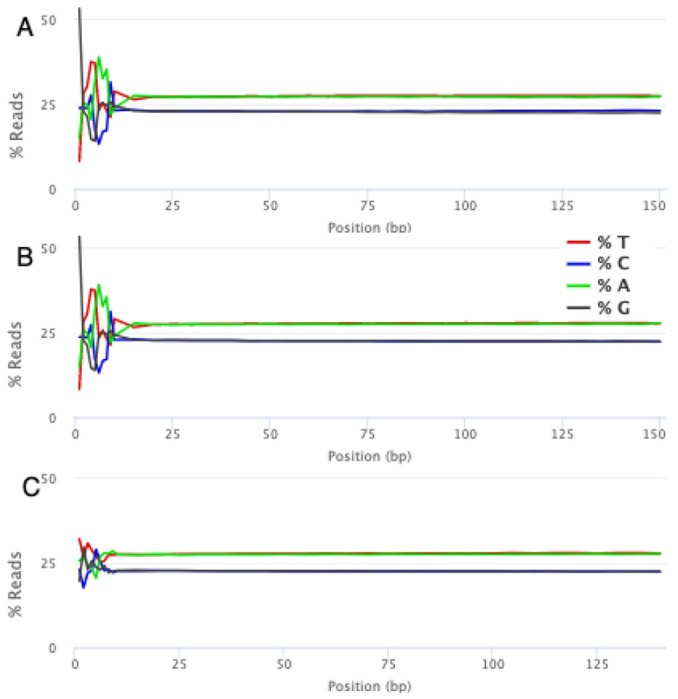
Per-base sequence content (PBSC) across the span of a short read is largely consistent, except in the first 9 bases of the read in the raw dataset and after a single round of fastp trimming. (A) PBSC in the raw dataset. (B) PBSC in the dataset after a single round of trimming. (C) PBSC in the dataset after the first 9 bases were further trimmed off.

From both samples, the first two technical replicates retained approximately 94% of the reads and 91% of the bases after BayesHammer error correction (Nikolenko et al. (2013)), whereas the last technical replicate only retained 76% and 71% respectively (Table 2 for sample 1, Supp. Table 1 for sample 2). The largest reduction in dataset size occurred at the fastp low-quality read removal step, followed by BayesHammer error correction because reads are removed in pairs, even if only one fails quality filtering. Upon visual inspection of the MultiQC reports (Ewels et al. (2016)), we found that across all technical replicates, even after running the full quality control module, the per-base sequence content was very uneven for the first 9 bases (and for replicates 1 and 2, the last 4 bases as well) (Figure 1). This could potentially be due to the fact that the sequencing libraries were fragmented using transposases which intrinsically biases the read start positions. While trimming does not address the start positions, the fact that these bases were also associated with lower sequence quality was grounds to remove them for a more cautious analysis strategy.

#### 2.2.4 Short-Read Taxonomic Classification

The short-read taxonomic classification Jupyter notebook semi-automatically compares replicates using several alpha and beta diversity metrics at all taxonomic ranks: simple numbers of species, Shannon entropy, Bray-Curtis dissimilarity and Jaccard difference.

**Fig. 2.**
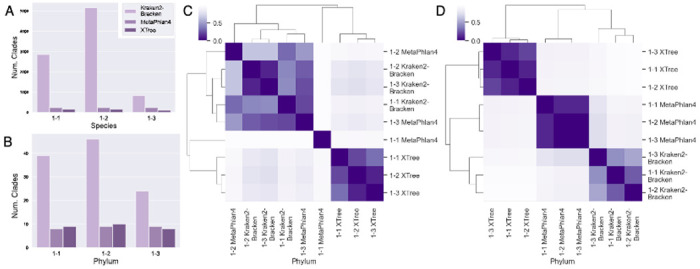
Genetic diversity metrics for a set of technical replicates (here, sample 1) can be visualized in several different ways. Alpha diversity as measured by the number of species at the ranks of (A) species and (B) phylum. Beta diversity between the nine sets of phyla discovered in each of the three technical replicates by each of the three classifiers as measured by (C) Bray-Curtis dissimilarity and (D) Jaccard difference.

Across all of the technical replicates, Kraken2-Bracken by far discovers the most taxa at every rank (Wood et al. (2019); Lu et al. (2017)), and XTree is the generally the most restrained (Figure 2A,B, only species and phylum shown) (GabeAl (2022)). Furthermore, the vast number of discovered taxa are not universal, though this is likely skewed by Kraken2-Bracken’s rate of discovery. For example, out of the 7,991 species discovered by all three algorithms across all three of sample 1’s replicates, there were 7,134 unique species, indicating that at most, 857 (5.4%) species were discovered by one classifier in at least technical replicate, or vice versa. As we will demonstrate, the former case is more likely.

Since we tried three classification algorithms on three technical replicates, we calculated the Bray-Curtis and Jaccard differences between the taxa discovered by all nine classifier-technical replicate combinations to explore discrepancies between classification algorithms. At the species level (Supp. Figure 1C), there are no trends in Bray-Curtis dissimilarities (which are calculated between the relative abundances of shared taxa) whereas at the phylum level, combinations are strongly clustered by the classification algorithm as opposed to the technical replicate of origin (Figure 2C). This tendency to cluster by algorithm is even stronger with Jaccard differences (Figure 2D), which only takes into account presence-absence. Only 29 species (0.41%) of unique species and 2/59 phyla (3.4%) were universally discovered. At the phylum level, only Firmicutes and Proteobacteria are common to all classifier-sample combinations and only 8/59 (13.6%) of phyla are found in a majority of the combinations. Similar results were observed in sample 2.

To triangulate the actual taxonomic composition of a sequencing dataset, it may be necessary to compare answers between several algorithms that each depend on different classification criteria. A highly automated analysis system where new algorithms can be easily added to a workflow and then compared to other algorithms with the same objective by either a core team or the algorithm’s developers is an invaluable tool for benchmarking as well as analysis studies.

#### 2.2.5 Short-read *De Novo* Assembly

Despite the fact that 1-3 is almost double the size of the next largest technical replicate dataset (Nayfach and Pollard (2015)), the number of contigs in 1-3’s *de novo* assembly is significantly smaller, the number of bases at least equal, and the average contig size is over twice as large. 1-3 had contigs up to 500 Kbp. The other technical replicates had contigs up to 50 Kbp with the exception of the second technical replicate of the second sample, which only has contigs up to 20 Kbp long. These are on the order of 1-2% of the size of the average human gut microbial genome size (Nayfach and Pollard (2015)). For all six technical replicates however, only a small fraction of the assemblies are of contigs larger than 20 Kbp (0.49% at maximum), even if the raw number is large.

When considered in concert with the short-read taxonomic classification results, it is interesting to note that although 1-3’s *de novo* assembly size was larger, it is unlikely to contain sequence information from a larger range of taxa. Across all classification algorithms, the smallest number of taxa discovered at every rank was found in 1-3. Thus, at this stage of analysis, it is more likely that the *de novo* assembly of 1-3 contains more breadth and/or depth of coverage of taxa in the gut microbiome sample, as opposed to a larger number of taxa.

Even so, the vast majority of contigs in all *de novo* assemblies are extremely small and fall below the size cutoffs of most MAG binning algorithms. Smaller contigs are more likely to contain low-quality bases and have irregular coverage (Hofmeyr et al. (2020)), though they are also more likely to originate from low-abundance organisms (Vicedomini et al. (2021)). To retain as much sequence information possible due to a limited (*de novo* assembly size), this visual assessment informed our selection of a minimum contig cutoff for the subsequent binning step (Table 3). For all binning algorithms except MetaBAT2 (Kang et al. (2019)) (which has a hard minimum of 1500 bp), we selected 1000 bp.

#### 2.2.6 MAG Binning and Quality Checking

**Fig. 3.**
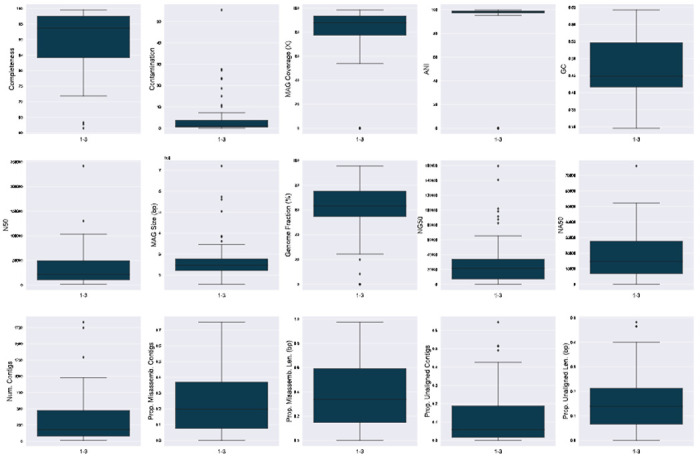
MAG quality-checking summary statistics for refined bins reconstructed from the 1-3 dataset. In order, from left to right then top to bottom: completeness, contamination, MAG coverage, ANI, GC (%), N50, MAG size, genome fraction (%), NG50, NA50, total number of contigs, proportion of contigs that are misassembled, proportion of sequence bases in misassembled contigs, proportion of contigs that do not align to the reference genome, proportion of sequence bases in unaligned contigs. The last 11 statistics were generated by QUAST.

**Fig. 4.**
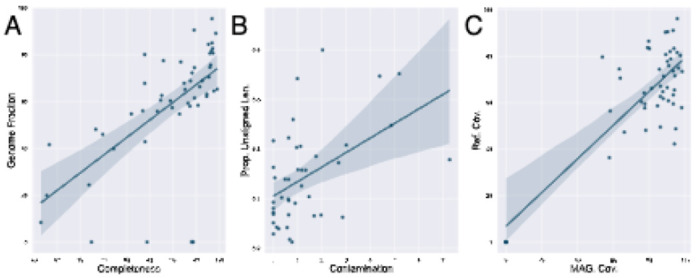
(A) Completeness and genome fraction, (B) contamination and proportion of sequence bases in unaligned contigs, and (C) MAG coverage (i.e.: relative abundance) and reference genome coverage are all positively correlated but with significant variance, especially when completeness and relative abundance are low and contamination is high.

With completeness ≥90% and contamination ≤5, 52.9% and 63.5% of the MAGs in samples 1’s and 2’s technical replicates would be considered high-quality MAGs according to MIMAG standards (Bowers et al. (2017)). The median completeness in both replicates are high- 93.7 and 94.3 respectively (Figure 3 row 1 column 1). However, the genome fraction of classifiable MAGs, which is the proportion of the species reference genome that the MAG was able to map to- is significantly lower by a larger margin- 65.8% and 77.2% (Figure 3 row 2 column 3). This aligns with Nelson et al.’s and Meziti et al.’s previous discoveries that marker-gene-based assessments of MAG completeness are frequently misleading estimates of the actual totality of an organism’s gene content (Figure 4A) (Nelson and Mobberley (2017); Meziti et al. (2021)). Furthermore, the median contamination of 1-3- 1.0- indicates that there is at least one more copy of each single-copy marker genes in the MAG (Figure 3 row 1 column 2). However, the median proportion of the MAG that is unaligned bases (bp) is 14.2% (Figure 3 row 3 column 5), is on the order of kilobase-pairs and thus generally underestimated by contamination (Figure 4B). Similar results were discovered in sample 2 (Supp. Figure 3).

Recall that only a small fraction of contigs larger than 20 Kbp (0.49% at maximum). The average size of a contig in each MAG in 1-3 is 13.9 Kbp. The median NA50 of the classifiable MAGs is 15.5 Kbp (Figure 3 row 2 column 5), indicating that most of the assemble-able and mappable content of a MAG is in a very small number of large contigs. The rest of the associated reference genome is likely hard to assemble and may have different sequence properties than the the rest of the contigs, supporting the use of BLAST as a strategy rather than a secondary binning strategy, which will likely rely on sequence similarity metrics such as tetranucleotide frequencies. Similar trends can be observed in 2-3.

Generally, there is higher read coverage of a MAG than the reference genome of its species classification. Since the median average nucleotide identity between a MAG and the reference genome of its classified species (if available) was 98.34 (Konstantinidis and Tiedje (2005)) with minimal standard deviation (range: 95.18-99.81, (Figure 3 row 1 column 4), the inferred MAGs are likely either a specific substrain of the associated species, or a mixture of strains (Figure 4C). To further explore strain heterogeneity within a MAG, a module designed for variant analysis and/or strain inference is required.

For the first two technical replicates of both samples, there were at most two MAGs generated after bin refinement by DAS Tool (Sieber et al. (2017)). It is likely each MAG’s contig composition was not stable enough for DAS Tool to confidently associate sets of contigs with each other. One analysis option could an assessment of the gene content of the assemblies, as DAS Tool relies on the presence of single-copy genes to assess MAG quality (Sieber et al. (2017)). Another option could be to use another bin refinement tool in place of DAS Tool to determine whether the failure to refine is an intrinsic problem of the bins generated by the upstream algorithms, or an incompatibility of the refinement algorithm itself with the sequence content of the dataset.

As demonstrated above with the MAG quality-checking and MAG generation failures, fully automated compressed summary statistics reports are essential to large-scale dataset analysis to extract relationships between both reference-based and reference-free statistics for reliability assessments.

#### 2.2.7 Computational Resources Used

If run as an end-to-end workflow, all five modules can be used to efficiently process the sequencing data in sample 1 in approximately 1 day. Similar times were achieved with sample 2.

## 3 Discussion

We have developed a modular metagenomics analysis system that is i) designed for integrated multi-analysis step data exploration and ii) automated tool benchmarking and comparisons. The system’s architecture makes it possible to run several tools as a discrete single-input to singleoutput unit for highly study-specific downstream steps (ex. raw sequencing reads to quality-filtered sequencing reads) or dozens of tools in a full end-to-end or multi-pronged workflow (ex. raw sequencing reads to short-read classification as well as MAG reconstruction) with an easy-to-install procedure and compute system compatibility.

The modular architecture allows for future expansions of both within-module utilities by way of adding new Snakemake rules, and entirely new modules thanks to the standardized module and working directory structures, which make it easy for core and external developers to ramp into study-specific customizations. The computation ecosystem of the modular system’s analytic capabilities is under ongoing expansion with many modules that are currently under active development include Oxford Nanopore long-read preprocessing, gene cataloguing, and metaviral/phage discovery. Future extensions for new avenues of investigation that have been demonstrated with existing one-click pipelines include: long-read and hybrid short-long assembly, multi-sample coassembly, variant and strain analysis, metatranscriptomics, and functional and pathway annotation.

## Supplementary Material

Supplement 1

## Figures and Tables

**Table 1.** Each feature in the module system was designed to maximize scalability (in terms of dataset size as well as system distribution), portability (i.e.: compatibility with existing analysis environments), ease of use, and analysis transparency and reproducibility.

Feature	How does it facilitate best-practices?
1) Standardized module template	• **Ease of use**: Every single module in the system has the same structure as described in Box 2.1.1, which makes it extremely easy to i) learn how to use existing modules, ii) customize existing modules with new tools and rules, and iii) make completely new modules. • The module template is a Cookiecutter (Cookiecutter (2022)) that can be used to start new modules from scratch in a few minutes. • **Scalability**: Thanks to a fully automated Cookiecutter-based template, the entire system is extremely flexible and can easily integrate developments in the metagenomics field. New modules for custom purposes easily be set up with the template and connected to existing modules in the system. For example, a hybrid short-long read assembly module can use as inputs the output samples.csv files from short- and long-read preprocessing.
2) Each module is a self-contained analysis unit	• Soft pauses are built into the end of every module so that the user can visually inspect the intermediate output and apply additional analysis steps that may not have been apparent at the outset. • **Portability**: Each module can be downloaded and set up individually. For example, if a user just wants to do short-read taxonomic classification, they only need the short-read preprocessing and classification modules and do not need to set up anything MAG-related.
3) Click-based Python command-line interface 4) Standardized input-output file format	• Snakemake’s API is unwieldly for coordinating multiple command-line parameters and computational resources, not to mention restarting and rerunning existing jobs. The intuitive Python-based command-line wrapper internally communicates with Snakemake’s API and allows users to interact with an easily operated interface. • **Ease of use**: The interface eliminates the need to write long, unwieldy, error-prone Snakemake commands. • The input and output of every module is always in a standardized samples.csv format that encodes the locations of input or output data for each sample in the dataset. • **Scalability**: This simple file format is the glue that allows modules to be joined together into whatever complexity of workflow the user needs.
5) Standardized working directory structures	• **Ease of use**: Similar to the standardized module directory, once the user is familiar with navigating the intermediate and output files of one module, all others are equally navigable. • **Transparency and reproducibility**: Intermediate and output reports as well as job logs are located in predictable and intuitive directory structures. If jobs fail, finding the error, whether it is due to computational under-resourcing or incorrectly set parameters, is straightforward.
6) Summary statistics of module outputs for accessible first-pass interpretations	• **Scalability**: Large datasets can be difficult to analyze without intuitive summaries. For example, the short-read taxonomic classification module comes with the taxa discovered by each of three classification algorithms as well as their estimated relative abundances in standardized CSVs for easy comparisons. • **Portability, transparency, and reproducibility**: By providing summaries at the end of each module, users can document every step of their analysis parsimoniously without having to keep every single large intermediate file.
7) Jupyter notebook-based semi-automated visualization of summary statistics	• **Ease of use and scalability**: While summary statistics are useful for documentation, visualizations are a more intuitive way to understand dataset outputs, especially large datasets with many samples that need to be compared.
8) Module-independent files for parameter and computational resource settings	• **Scalability**: While a default set of parameter and resource values are provided with the sample config YAML, users can set different parameter and resource profiles depending their data. • **Portability, transparency, and reproducibility**: To share the parameter and resource settings used in their analyses, users need only share these two text files. • **Ease of use**: Users only have to refer to these two text files to adjust parameters and resources without creating complex data structures on the command line.
9) Included test dataset and sample outputs from test dataset	• **Portability**: The user can verify proper module setup, as well as visually inspect the proper intermediate and output file formats and work directory to gain mechanistic familiarity the purpose of each step in the module.
10) Conda-based environment setup with integrated YAMLs	• **Scalability**: Instead of re-installing existing Conda environments into every analysis working directory, Conda environments are built directly into the module directory only once. • **Portability and ease of use**: Complex conflict-free environments are deployed along with the module workflow code, and can be easily set up using standard Conda commands. • **Transparency and reproducibility**: Environments are standardized and auto-documented for version consistency.
11) Slurm job scheduler integration	• **Scalability**: Allowing modules to be run with job scheduler assistance helps users with access to HPCs to parallelize running rules within the module.

**Table 2.** Sample 1 sequencing dataset size reductions after each step of the short-read preprocessing module.

Replicate	QC Step	Num. Reads	Prop. Raw Reads	Size (bp)	Prop. Raw Bases	Mean Read Len. (bp)
1-1	Raw dataset	74754948	1.000	11258026332	1.000	150.6
1-1	Low qual. removal	70618640	0.945	10234827951	0.909	144.93
1-1	Adapter removal	70618640	0.945	10234796232	0.909	144.93
1-1	Host read removal	70614144	0.945	10234151423	0.909	144.93
1-1	Error correct.	70451148	0.942	10209681737	0.907	144.92
1-1	Retrimming	70341566	0.941	9278908958	0.824	131
1-2	Raw dataset	59182340	1.000	8912502768	1.000	150.59
1-2	Low qual. removal	56219748	0.950	8188386269	0.919	145.65
1-2	Adapter removal	56219748	0.950	8188361828	0.919	145.65
1-2	Host read removal	56208930	0.950	8186815427	0.919	145.65
1-2	Error correct.	56000336	0.946	8155457319	0.915	145.63
1-2	Retrimming	55917268	0.945	7416057194	0.832	132
1-3	Raw dataset	215351304	1.000	32302695600	1.000	150
1-3	Low qual. removal	164704400	0.765	24331210934	0.753	147.73
1-3	Adapter removal	164704388	0.765	24331202425	0.753	147.73
1-3	Host read removal	164702372	0.765	24330911951	0.753	147.73
1-3	Error correct.	163944036	0.761	24217279298	0.750	147.72
1-3	Retrimming	162684468	0.755	22564731304	0.699	139

**Table 3.** We selected a minimum contig size cutoff for MAG reconstruction partially based on the amount of sequence information that would be retained in the dataset. In the third and fourth columns, the proportion of the total number of contigs and sequence bases that would be retained respectively are in brackets.

Replicate	Min. Contig Size (bp)	Num. Contigs Retained	Num. Bases Retained
1-1	1000	53726 (0.031)	82742700 (0.168)
1-1	2500	3547 (0.002)	13773658 (0.028)
1-2	1000	44259 (0.026)	68114022 (0.150)
1-2	2500	3038 (0.002)	11071502 (0.024)
1-3	1000	64058 (0.126)	296587793 (0.660)
1-3	2500	23861 (0.047)	235809940 (0.525)

**Table 4.** Real time required to process all technical replicates of sample 1 where 60 CPU (Intel(R) Xeon(R) Gold 6240R) were made available, except for the short-read taxonomy step and MAG quality-checking, where 40 CPUs were. MAG quality-checking was only run o technical replicate 3of both samples 1 and 2.

Module	Time (hh:mm)
Short-read preprocessing	7:49
Short-read taxonomy	6:34
Short-read assembly	3:33
MAG binning	14:51
MAG quality-checking	1:13

## Box

workflow/: Contains all of the module’s functional code and scripts from external tools that are under an open-source license.

module_slug.py: The Click-based command-line interface that wraps a Snakemake-based workflow.
Snakefile: Workflow that itself wraps Snakemake rules, which call external algorithms or internal functions (utils.py) to process data.
utils.py: Internal functions such as dataset ingestion and working directory setup.
ext/: External scripts and smaller data files required by algorithms used in the workflow.

configs/: Contains all settings files.

parameter.yaml and resource.yaml: Instead of relying on Snakemake’s dictionary-based system of specifying parameters and resources on the command line, algorithm parameters and computational resource allocations have been unified into single YAML files. Users can: i) use the default YAMLs provided, ii) modify the values in the YAMLs according to their dataset properties, or iii) make multiple copies of th YAMLs to analyze datasets of different sizes and biological origins.
samples.csv: A standardized input file where each row corresponds to a sample. The first column is always the sample’s name, and every column thereafter contains paths to that sample’s data.
conda/: Conda environment recipes. The main environment is always called module_slug.yaml and should be set up immediately after cloning the module to a local directory. All other Conda environments will be set up the first time the workflow is run into a directory called conda_envs/ in the module’s top-level directory.
sbatch/: A Snakemake profile that enables the module’s rules to be run using the Slurm job scheduler.

test_data/: Test data to ensure proper module setup, and an example work directory to demonstrate its structure, and input, intermediate, and output file formats.

dataviz.ipynb: Guided and semi-automated data visualization of the module’s final output using Jupyter notebook. Users can use the built in graphing functions as is or customize as needed.
