# CAMP: A modular metagenomics analysis system for integrated multi-step data exploration

**DOI:** 10.1101/2023.04.09.536171

**Published:** 2024-09-14

**Authors:** Lauren Mak, Braden Tierney, Cynthia Ronkowski, Rodolfo Brizola Toscan, Berk Turhan, Michael Toomey, Juan Sebastian Andrade Martinez, Chenlian Fu, Alexander G Lucaci, Arthur Henrique Barrios Solano, João Carlos Setubal, James R Henriksen, Sam Zimmerman, Malika Kopbayeva, Anna Noyvert, Zana Iwan, Shraman Kar, Nikita Nakazawa, Dmitry Meleshko, Dmytro Horyslavets, Valeriia Kantsypa, Alina Frolova, Andre Kahles, David Danko, Eran Elhaik, Pawel Labaj, Serghei Mangul, The International MetaSUB Consortium, Christopher E. Mason, Iman Hajirasouliha

**Affiliations:** 1 Tri-Institutional Computational Biology & Medicine Program, Weill Cornell Medicine of Cornell University, NY, USA; 2 Institute for Computational Biomedicine, Weill Cornell Medicine of Cornell University, NY, USA; 3 Department of Physiology and Biophysics, Weill Cornell Medicine of Cornell University, NY, USA; 4 Titus Family Department of Clinical Pharmacy, University of Southern California, CA, USA; 5 Department of Quantitative and Computational Biology, University of Southern California, CA, USA; 6 Małopolska Centre of Biotechnology, Jagiellonian University, Kraków, Poland; 7 Department of Biochemistry, Institute of Chemistry, Universidade de São Paulo, São Paulo, Brazil; 8 Natural Resource Ecology Laboratory, Colorado State University, CO, USA; 9 Two Frontiers Project, State, Country; 10 Broad Institute of MIT and Harvard, MA, USA, Mathematics Institute, University of Warwick, Coventry, UK; 12 Nazarbayev Intellectual School of Physics and Math, Almaty, Kazakhstan; 13 School of Molecular and Theoretical Biology, Tartu, Estonia; 14 Institute of Molecular Biology and Genetics, NASU, Kyiv, Ukraine; 15 Kyiv Academic University, Kyiv, Ukraine; 16 Taras Shevchenko National University, Kyiv, Ukraine; 17 ETH, Zurich, Switzerland; 18 Biotia, NY, USA; 19 GeoSeeq Foundation, NY, USA; 20 Department of Biology, Lund University, Sweden; 21 WorldQuant Initiative for Quantitative Prediction, Weill Cornell Medicine of Cornell University, NY, USA, The Feil Family Brain and Mind Research Institute, Weill Cornell Medicine of Cornell University, NY, USA; 23 Englander Institute for Precision Medicine, Weill Cornell Medicine of Cornell University, NY, USA

**Keywords:** Metagenomics, Workflow Management Systems, Taxonomic Classification, De Novo Assembly, Metagenome-Assembled Genomes, Metaviromics, Gene Cataloguing

## Abstract

**Motivation:**

Computational analysis of large-scale metagenomics sequencing datasets have proven to be both incredibly valuable for extracting isolate-level taxonomic, and functional insights from complex microbial communities. However, due to an ever-expanding ecosystem of metagenomics-specific methods and file-formats, designing studies which implement seamless and scalable end-to-end workflows, and exploring the massive amounts of output data have become studies unto themselves. One-click bioinformatics pipelines have helped to organize these tools into targeted workflows, but they suffer from general compatibility and maintainability issues.

**Methods:**

To address the gap in easily extensible yet robustly distributable metagenomics workflows, we have developed a module-based metagenomics analysis system: ”Core Analysis Metagenomics Pipeline” (CAMP), written in Snakemake, a popular workflow management system, along with a standardized module and working directory architecture. Each module can be run independently or conjointly with a series of others to produce the target data format (ex. short-read preprocessing alone, or short-read preprocessing followed by *de novo* assembly), and outputs aggregated summary statistics reports and semi-guided Jupyter notebook-based visualizations.

**Results:**

We have applied CAMP to a set of ten metagenomics samples to demonstrate how a modular analysis system with built-in data visualization at intermediate steps facilitates rich and seamless inter-communication between output data from different analytic purposes.

**Availability:**

The module template as well as the modules described below can be found at https://github.com/MetaSUB-CAMP.

## Introduction

Metagenomics refers to sequencing-based investigation of all microorganisms within an environmental or host-associated microbial community. These communities are typically complex mixtures of dozens to hundreds of species. Since many organisms are yet to be cultured for targeted sequencing, the taxonomic and functional properties of the community must be recovered from aggregated shotgun metagenomic sequencing data. This wet-to-dry workflow has yielded some incredible insights into the ecology and evolution of samples taken from environments [Almeida et al., 2019, Danko et al., 2021, Kadosh et al., 2020, Brito et al., 2019, Sierra et al., 2022].

The dry component of the metagenomics analysis workflow is responsible for processing sequencing data to extract study-specific taxonomic and/or functional information. Typically, the taxonomic unit of choice for downstream analysis is the metagenome-assembled genome, or MAG, a microbial isolate genome reconstructed from sequencing data. Alternative strategies involve classifying sequencing reads directly, or characterizing the functional content of the entire community through gene annotation of *de novo* assembled contigs [Quince et al., 2017].

A central challenge of metagenomic analyses is the organization and application of multiple computational tools into workflows to extract biologically relevant and interpretable insights from raw sequencing reads [Ten Hoopen et al., 2017]. Due to the explosion of large-scale sequencing datasets, with samples numbering from the dozens to the hundreds, there is a field-wide need to develop workflows that are straightforward to test, maintain, and reuse, all the while capable of generating reproducible results [Djaffardjy et al., 2023]. While there are a vast array of open-source tools available, many of them are not accessible online, easy to install, or even installable at all [Mangul et al., 2019]. A widespread problem in bioinformatics is the short life span of tools, Kern et al. [2020] reported that of the 2,396 web tools they surveyed in a 133-day test frame, 31% of tools were always working, 48.4% occasionally and 20.6% never worked. Open-source package management systems such as Conda, containerization systems such as Docker and Singularity, and workflow management systems, such as Nextflow and Snakemake, have partially addressed these challenges. Dependency conflicts and operating incompatibilities are still major problems when multiple tools need to co-exist in the same environment [ana, 2020, Merkel, 2014, Kurtzer, 2018]. Of the tools that are easy to install and use, only a smaller subset have been benchmarked on realistically simulated datasets, making it difficult to evaluate their strengths and weaknesses [Sczyrba et al., 2017, McIntyre et al., 2017, Zhang et al., 2022, Meyer et al., 2018], and there exists a need for a comprehensive gold-standard datasets for tools comparison. While seemingly promising, all-in-one workflows harbor a set of challenges. Because they wrap multiple tools, there are many dependent points of failure at both installation and run time. Workflows that do not support workload managers such as Slurm and instead rely on containerization are not widely usable on high-performance compute (HPC) clusters with root-access restrictions. Many workflows balance ease of use with parameter complexity by heavily favoring the former and masking the parameter ranges of the encapsulated tools, usually hard-coding them as the default value. All-in-ones tend to be developed and maintained by a single laboratory and are limited in terms of end-user customization potential. Easy-to-install workflows with well-chosen default settings can be suitable for researchers familiar with basic Unix commands. However, such workflows can be analytic black boxes, taking end-users from input to desired output with a one-line command. The intermediate results are implicitly obscured, preventing the end-user from integrating their domain expertise and allowing manual curation to play a role in data cleaning and analysis. As has been demonstrated, in-depth intermediate results exploration is an integral part of ensuring MAG quality, especially in the reconstruction of circularized and (near-)complete microbial isolate genomes [Chen et al., 2019, Moss et al., 2020].

An alternative way to conceptualize metagenomics analyses is to design workflows themselves as an interconnected suite of modules. To that end, we have designed a modular metagenomics analysis system comprised of several self-contained workflows and semi-guided visualizations, called modules. Each module is designed to complete a single analytic task (ex. *de novo* assembly), accepting a standardized input format (ex. CSV of paths to FastQ files) generated by antecedent modules (ex. read preprocessing), and generating a standardized output format(s) (ex. CSV of paths to assembled contigs, summary statistics report). While each module wraps a different set of analytic tools, every module shares a common command-line interface, module directory structure, and working directory structure, facilitating intuitive data navigation by the user as well as numerous customization opportunities. By wrapping Snakemake internals using this command-line interface, our modular workflow facilitates the following analytic utilities:

- **‘Set menu’-style computing to ‘a la carte’-style study design**: Switching from one-click pipelines to a modular analysis system allows the user to assemble the unique workflow for their specific study by downloading only the necessary modules.
- **Built-in soft pauses at the end of each module**: At the conclusion of each module, or ‘step’ in the workflow, the user can explore various semi-automated visualizations of their analytical results and apply their own knowledge base to enhance downstream analyses. This includes modifying downstream parameters from their default values.
- **Modules as benchmarking and comparison meta-tools:** A module can serve as a benchmarking ‘sandbox,’ akin to the Snakemake pipeline described by [Orjuela et al., 2022], where new methods can be seamlessly incorporated into a workflow and subsequently compared to other methods with similar objectives.
- **Compressed summary statistics tables**: The standardized output dataframes can serve as inputs for downstream machine learning ingestion.
- **Semi-guided visualizations**: Visualizations are essential for large-scale dataset analysis, hypothesis generation, and reliability assessments.
- **Module template for future expansions**: Each module is based on a standard directory template. New modules for new analysis purposes can be easily set up from scratch using the cookiecutter command within a few hours. This process includes creating Conda environments and generating module-specific parameter and resource files.

## Methods

**Fig. 1.**
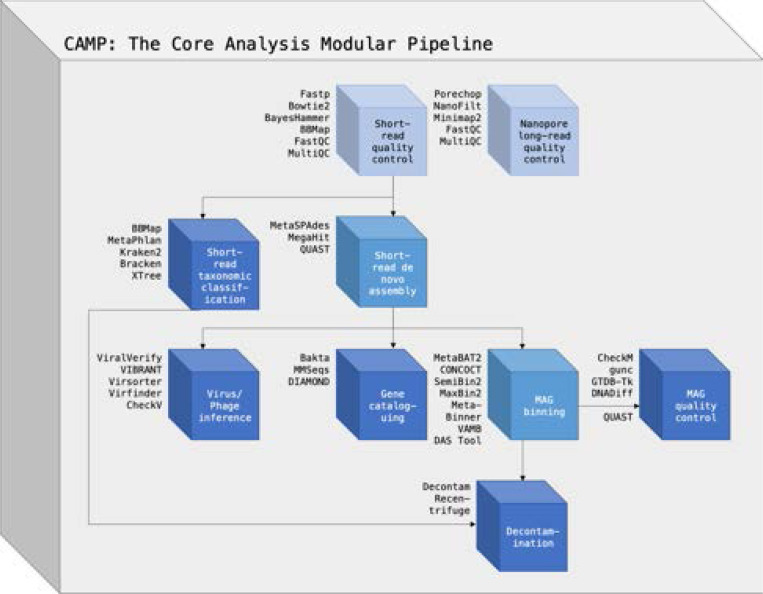
An overview of the available metagenomics analysis modules in the Core Analysis Modular Pipeline (CAMP). All modules share the same internal architecture, but wrap a different set of algorithms (shown to the left of each box) customized to its particular analysis goals. Modules that are typically the beginning of analysis projects are coloured light blue, modules that are typically intermediate steps are coloured medium blue, and modules that are typically terminal analysis steps are coloured dark blue.

Each module is a GitHub repository containing core components organized in a standardized directory structure. This ensures consistency across the system, including the module directory (Box 1), working directory, parameterization, and input/output architecture. The core components include the Snakefile, utils.py, ext/directory, parameter.yaml, and resource.yaml, which are further customized for the module’s specific purpose. The contents of each module, as well as the encapsulated tools, are described in the Supplementary References. The design features of the modular system are outlined in Table 1, and available analysis modules are further described in Figure 1.

### Comparison of CAMP Workflow and MetaSUB Analysis Methods

The methodology of the original MetaSUB study was designed to profile the taxonomic and functional content of nearly 5,000 urban microbiome samples from around the world [Danko et al., 2021], whereas this study was tailored to demonstrate the technical features of CAMP using ten samples. An example of the procedural differences and their purposes is described in the Supplementary Materials. While the results are not intended to match, it is nonetheless instructive to compare them.

### Workflow Management System

While Nextflow [Di Tommaso et al., 2017] and Snakemake [Mölder et al., 2021] are both popular choices as workflow management systems for computational biology analysis, we opted for Snakemake due to its general readability and accessibility, particularly accustomed to using Python. Snakemake’s syntax is minimal and resembles standard Python, making workflows easy to understand, develop, expand, and troubleshoot. Although working with Snakemake’s wildcards may pose a learning curve, its directives (e.g., output, log) and ability to specify file names explicitly generally result in output directories that are easy to navigate and conducive to workflow reruns. Nextflow also offers many strengths as a workflow management system (WMS), with better support for cloud deployment natively as well as nf-core, a repository of curated analysis pipelines developed by the community at large. Currently, there are seven pipelines for metagenomics sequencing data with analysis goals that overlap with CAMP. Note, that the nf-core metagenomics pipelines are not currently interoperable.

### Environment Management

We have opted not to implement the modular system with Docker [Merkel, 2014] or Singularity [Kurtzer, 2018], because many users of high-performance compute clusters (HPCs), lack root access privileges and thus cannot utilize containerized workflows. Instead, we have included conflict-free recipes in each module directory, each of which sets up a Conda environment directly. This allows the same environment to be used for all datasets processed using the module [ana, 2020]. To ensure reproducibility and interoperability within the module environment, each Conda recipe has hard-coded algorithm versions and is designed exclusively for Unix-based operating systems.

### Modular System Use-cases

The primary strengths of a modular system are its flexibility and extensibility, complemented by the interdependency of the tools. This architecture enhances accessibility and simplifies maintenance, enabling a longer lifespan. Such a design is accessible not only to the developers of the module template and core modules but also to anyone with fundamental command-line, Python, and Conda development skills. Below are a few example use cases:

1. A user possessing a short-read dataset and seeking to conduct an initial analysis with short-read taxonomic classification can download two modules: ”short-read preprocessing” for the removal of low-quality and erroneous bases, and ”short-read taxonomic classification” for the generation of a unified report consolidating discovered taxa from three classification tools. Simplifying the learning process, the user interfaces with a single command-line interface (CLI), which remains consistent across all modules.
2. The user, intending to expand their analysis to include gene annotation, can effortlessly do so by downloading and executing two additional modules: ”short-read assembly” and ”gene cataloging.” No additional onboarding time is necessary in terms of interface learning. Moreover, the input for short-read assembly remains consistent with that of short-read taxonomic classification, utilizing the samples.csv file containing paths to preprocessed short reads.
3. The user, aiming to broaden their analysis to include MAG reconstruction, can seamlessly achieve this by downloading the binning and MAG quality-checking modules. Utilizing the output of short-read assembly, specifically the samples.csv file containing paths to the *de novo* assemblies, as their input, no additional onboarding time is required to learn new interfaces.
4. A user who has developed a new short-read taxonomic classification tool can leverage the existing short-read taxonomic module as a sandbox environment. They can write a custom Snakemake rule to execute their tool, implement a function to harmonize the output format with the existing merged report, and conduct benchmarking analyses to assess the strengths and weaknesses of their tool.
5. A user aiming to study extra-chromosomal elements, given the absence of an existing module for this purpose, can create a new blank module utilizing the Cookiecutter template. They can then populate it with the requisite Snakemake rules and customize the remaining demo configurations to suit their specific objectives. Leveraging the standardized input-output format ensures seamless integration of this new extra-chromosomal elements module with the broader module system, facilitating reuse by both the developer and the wider research community.

### Currently Available Modules

There are ten modules available, and seven are used in this proof-of-concept analysis to accomplish one of three purposes: i) short-read taxonomic classification, ii) MAG reconstruction and quality-checking, and iii) virus and phage inference from short-read sequencing datasets. There are several modules currently under active development, including Oxford Nanopore long-read preprocessing, mOTUs, and decontamination. The algorithms included for each module were chosen based on previously documented performance in benchmarking studies, as well as code stability and overall workflow compatibility.

### General-Purpose Analysis

#### Module 1: Short-read Preprocessing

Raw sequencing datasets are filtered for low-quality bases, low-complexity regions in reads, and extremely short reads using fastp [Chen et al., 2018]. Reads can optionally be deduplicated. Filtered reads are trimmed of adapters using Trimmomatic [Bolger et al., 2014]. If host read removal is selected, trimmed and filtered reads are mapped using Bowtie2 and Samtools with the ‘very-sensitive’ flag to the host reference genome (here, the human reference genome assembly GRCh38), and mapped reads removed [Langmead and Salzberg, 2012, Li et al., 2009]. As a last-pass, BayesHammer or Tadpole is used to correct sequencing errors [Nikolenko et al., 2013, Bushnell, 2014]. FastQC and MultiQC are used to generate overviews (ex. parameters such as per-base quality scores, sequence duplication levels) of processed dataset quality [Andrews, 2010, Ewels et al., 2016].

#### Module 2: Short-read Assembly

The processed sequencing reads can be assembled using MetaSPAdes (with optional flags for metaviral and/or plasmid assembly also available), MegaHIT, or both [Nurk et al., 2017, Li et al., 2015]. For the purposes of this study, only MetaSPAdes was used. The assembly is subsequently summarized using MetaQUAST [Mikheenko et al., 2015].

### MAG Inference and Quality-Checking

#### Module 3: MAG Binning

Processed sequencing reads are mapped back to the de novo assembled contigs using Bowtie2 and Samtools. This read coverage information, along with the contig sequences themselves, are used as input for the following binning methods: MetaBAT2, CONCOCT, SemiBin, MaxBin2, VAMB, and MetaBinner [Kang et al., 2019, Alneberg et al., 2014, Pan et al., 2023, Nissen et al., 2021, Wu et al., 2015, Wang et al., 2023]. The sets of MAGs inferred by each algorithm are used as input for DAS Tool, an ensemble binning methods, to generate a set of consensus MAGs scored based on the presence/absence of single-copy genes (SCGs) [Sieber et al., 2017]. Some of the contig pre-processing scripts were adapted from the MAG Snakemake workflow [Saheb Kashaf et al., 2021].

#### Module 4: MAG Quality-Checking

The consensus refined MAGs are quality-checked using an array of parameters. CheckM2 calculates completeness, which is based on the number of lineage-specific marker gene sets present in a MAG, and contamination, which is the number of over-represented multiple copies of a marker gene in a MAG [Chklovski et al., 2023]. gunc is also used to assess contamination [Orakov et al., 2021]. MAGs are classified using GTDB-Tk, which relies on approximately calculating average nucleotide identity (ANI) to a database of reference genomes [Chaumeil et al., 2019]. For MAGs with a species classification, their contig content is compared to the species’ reference genome and genome-based completion, misassembly, and non-alignment statistics calculated using QUAST [Gurevich et al., 2013].

Our modified quality standards are based on the original MIMAG genome reporting standards [Bowers et al., 2017], Supp. Table 1). While we did not manually conduct assembly quality assessment or assess the presence of rRNA and tRNA genes, we incorporated the use of gunc’s clade separation score (CSS) as an additional and orthogonal indication of taxonomically-informed MAG gene content chimerism. gunc flags a genome as potentially contaminated if the CSS is larger than 0.45 at any taxonomic rank [Orakov et al., 2021].

### Other Analysis Goals

#### Module 5: Short-read Taxonomic Classification

The processed sequencing reads can be classified using MetaPhlan4, Kraken2/Bracken, and/or XTree [Wood et al., 2019, Lu et al., 2017, Blanco-Miguez et al., 2022, GabeAl, 2022]. All three tools were used here. To estimate the relative abundance of a taxon, MetaPhlan4 calculates marker gene coverage, Bracken calculates the proportion of reads assigned to a taxon with k-mer uniqueness-based scaling, and XTree estimates directly from unique k-mer proportions. Since each of these output reports are of different formats, the raw reports from each algorithm are standardized in format for easier comparisons downstream.

#### Module 6: Virus/Phage Inference

The processed sequencing reads are assembled with MetaSPAdes, and viral contigs are subsequently identified using the output assembly graph and ViralVerify [Nurk et al., 2017, Antipov et al., 2020]. Contigs containing putative viral genetic material are also identified using VIBRANT, VirSorter, and VirFinder [Kieft et al., 2020, Roux et al., 2015, Ren et al., 2017]. The aggregated lists of contigs from the three inference methods is dereplicated using VirClust [Moraru, 2023] and merged with the ViralVerify list, and the overall quality of the putative viruses is assessed using CheckV [Nayfach et al., 2020].

#### Module 7: Gene Cataloguing

Open reading frames (ORFs) are identified in the de novo assembly using Bakta, and clustered using MMSeqs [Schwengers et al., 2021, Hauser et al., 2016]. Genes are identified from these ORFs by alignment to the DIAMOND database to obtain the functional profile of the sample [Buchfink et al., 2014].

#### Module 8: Nanopore Long-Read Quality Control

Raw sequencing datasets are trimmed using PoreChop, and then low-quality bases are filtered out using NanoFilt [Wick, 2018, De Coster et al., 2018]. Host reads are optionally removed using Minimap2 [Li, 2018]. FastQC and MultiQC are used to generate overviews (ex. parameters such as per-base quality scores, sequence duplication levels) of processed dataset quality [Andrews, 2010, Ewels et al., 2016].

#### Module 9: Decontamination

The decontamination module is still under construction. A feature table of relative abundances (ex. operational taxonomic units (OTUs), taxa, metagenome-assembled genomes) is provided to Decontam and Recentrifuge, each of which estimates contamination from feature abundances either within or between samples respectively [Davis et al., 2018, Martí, 2019].

#### Module 10: mOTUs Profiling

The profiling module wraps mOTUs ([Ruscheweyh et al., 2021], which estimates the relative abundance of taxa from a short- or long-read sequencing dataset, and calls single-nucleotide variants from marker genes.

## Results

### Proof-of-Concept and Data

To demonstrate CAMP’s applicability for large-scale metagenomics analysis and inter-sample comparison, we have applied seven modules to a randomly selected set of 10 samples from the International Metagenomics and Metadesign of Subways and Urban Biomes (MetaSUB) dataset, which consisted of (at time of [Danko et al., 2021]’s publication) of 4,728 samples of microbiomes collected from urban transit system surfaces between 2015 – 2017. Sample collection, metagenomic DNA extraction, and sequencing information can be found in [Danko et al., 2021] and the GeoSeeq repository. The raw sequencing sequencing data can be found under the SRA accession ID PRJNA732392, as well as the GeoSeeq repository. The 10 randomly chosen samples were collected from subway train and transit station surfaces from three of the four boroughs of New York that are connected by subway transit lines-Manhattan, Queens, and Brooklyn (no samples from the Bronx were in the subset, Table 2). Most of the samples were obtained from transit station surfaces in the winter of 2014, and generally contain between 2 – 6 million paired-end reads.

### Short-Read Quality Control

**Fig. 2.**
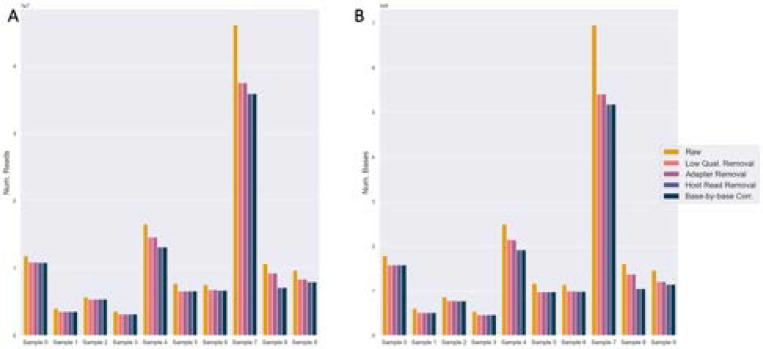
Quality control and preprocessing of short-read sequencing data. Counts of (A) reads and (B) bases retained after each preprocessing step across all samples. The steps include low-quality base trimming, adapter removal, host genome removal, and error correction.

Between 66.6 – 93.8% of short sequencing reads and 65.3 – 89.8% of sequencing bases were retained after low-quality base-trimming, adapter trimming, host (human genome GRCh version 38) genome removal, and error correction steps were applied to each of the 10 samples (Figure 2, Supp. Table 2). The largest reduction in dataset size occurred at the fastp low-quality read removal step, with host read removal by Tadpole also eliminating some sequence content (Figure 2A). No reads and bases were lost at the adapter step despite adapters being present in the pre-flight MultiQC check, indicating that all of the adapter bases were trimmed by fastp.

### Short-Read Assembly

**Fig. 3.**
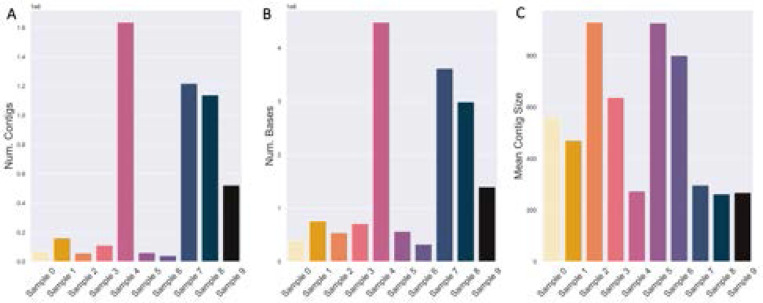
*De novo* assembly sizes generally correlate with short-read sequencing dataset sizes. (A) The number of contigs and (B) number of sequencing bases in each sample’s assembly, as well as the (C) mean contig size.

The distribution of assembly sizes mostly follows the distribution of short-read dataset size, except for Sample 4 (Figure 3A, Figure 2B). Although the size of Sample 7 is nearly 3X larger than sample 4 (in terms of both numbers of reads and bases), Sample 4 has the largest assembly by far in terms of both numbers of contigs and bases (Figure 3). MultiQC reported that Samples 4 and 7 contained 7.4% and 10.9% duplicated reads overall, which accounts minimally for the difference. Sample 4 may contain a much lower proportion of PCR duplicates. As shown later by both short-read taxonomic classification and MAG binning, sample 4 does not contain appreciably fewer species than sample 7. The largest, average, and median contig sizes are similar, though there is sizable variance in terms of assembly contiguity as measured by N50 (Supp. Table 3).

The vast majority of contigs in all *de novo* assemblies are extremely small and fall below the size cutoffs of most MAG binning algorithms (two examples in Table 3). Smaller contigs are more likely to contain low-quality bases and have irregular coverage [Hofmeyr et al., 2020], and are also more likely to originate from low-abundance organisms [Vicedomini et al., 2021]. To retain only high-quality sequencing information for binning, we selected a MAG binning minimum contig cutoff of 2500 base-pairs. Only a small fraction of the assemblies are of contigs larger than 50 Kbp (0.2% at maximum), which is approximately 1% of the size of the average bacterial genome [diCenzo and Finan, 2017].

### MAG Binning

**Fig. 4.**
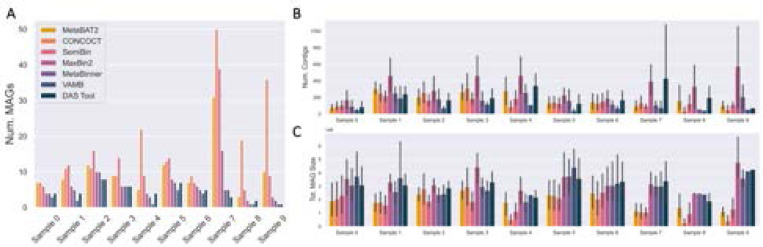
Most MAG binning algorithms infer a consistent number of MAGs, with the exceptions of samples 4, and 7 – 9 and VAMB. (A) Number of MAGs inferred by each binning algorithm across samples. (B) The numbers of contigs per (C) Total MAG size inferred for each sample and comparison across binners (bars indicate standard deviation).

Aside from VAMB and samples 7 – 9, the number of MAGs inferred by each binner (including the MAG inference aggregator and ensemble binner DAS Tool) was generally within a factor of two of each other (Figure 4A). In general, the more MAGs inferred by a binner, the smaller the inferred MAGs on average in terms of the numbers of sequencing bases (Figure 4A,C). The three binners that do not use single-copy marker genes to estimate the number of MAGs-MetaBAT2, CONCOCT, and SemiBin- tend to infer a larger number of MAGs (Figure 4A) [Kang et al., 2019, Alneberg et al., 2014, Pan et al., 2023, Nissen et al., 2021, Wu et al., 2015, Wang et al., 2023, Sieber et al., 2017]. SemiBin only uses single-copy marker genes to further partition MAGs with an average of > 1 copy per gene [Pan et al., 2023]. While MaxBin2 and MetaBinner generally inferred the same number of MAGs, MaxBin2 MAGs generally contain many more contigs but are similar in overall size (as counted by base pairs). This can be explained by MetaBinner’s stringent post-clustering contig reassignment step; MetaBinner uses a MetaWRAP-like consensus method to remove contigs that are not robustly associated with cluster centroids [Wang et al., 2023].

Despite having the largest assembly, only 4 MAGs were inferred from sample 4 by DAS Tool (Table 4). Furthermore, the size of the MAGs is approximately 2 Mbp, which is smaller than the average bacterial genome (Figure 4C) [diCenzo and Finan, 2017]. The largest number of MAGs was inferred from sample 2 (Table 4), which was the third smallest dataset and assembly (by the number of bases, Figure 2B, Figure 3B). However, sample 2 also had the largest average contig size, so more contigs were included in the binning process after applying a minimum size threshold of 2500 bp (Figure 3C). Unlike the original 2021 study, the number of identified MAGs did not correlate well with the number of reads [Danko et al., 2021].

Aside from VAMB, DAS Tool generally inferred the fewest MAGs, though they are usually the largest and similar to the average size of a bacterial genome (Figure 4C) [diCenzo and Finan, 2017]. In terms of overall assembly utilization, DAS Tool MAGs contained between 91.3 – 99.7% of usable bases in contigs >2500 bp.

### MAG QC

**Fig. 5.**
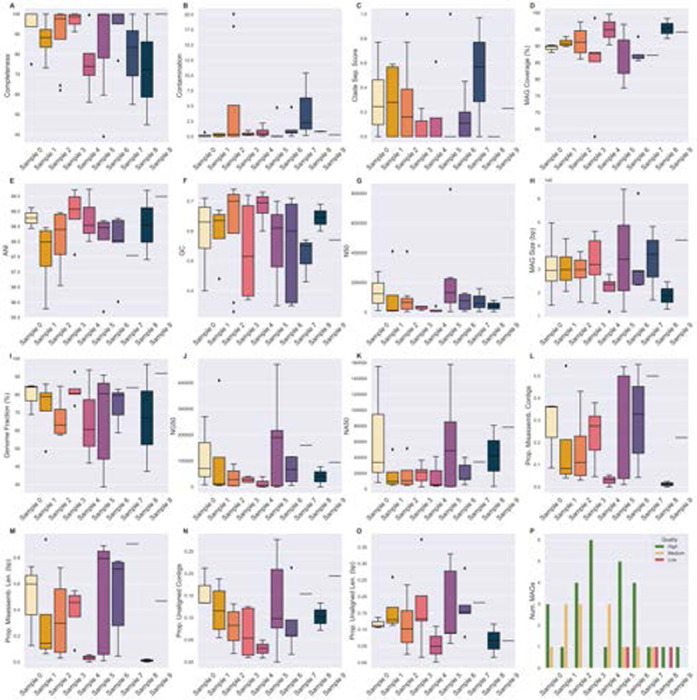
Quality assessment metrics of DAS Tool-inferred MAGs across all samples. The median of plot’s metric across all of the MAGs inferred from that sample is indicated with a line across the box. (A) Completeness, (B) contamination, (F) GC, (G) N50, and (H) MAG size were reported by CheckM2. (C) Clade separation score was reported by gunc. (D) MAG coverage by the GTDB-Tk classified reference genome was reported by dnadiff. Metrics (I - O) were reported by QUAST comparing each MAG to the GTDB-Tk classified reference genome.

Most of the inferred MAGs (27/44, 61.3%) across all samples were high-quality, according to the modified MIMAG standards described in the Methods section (Figure 5P). Of the low-quality 3/44 (9.2%) MAGs, 2/3 had low CheckM2-estimated contamination and gunc-estimated clade separation score (CSS) and were still classifiable to the species level. The remaining low-quality high contamination MAG (CheckM2: 10.81, gunc: 0.97) was classified to the genus Pseudomonas. Given the high CSS, the MAG may be a chimeric bin of contigs from multiple genera in the Pseudomonadaceae family. CheckM2-estimated completeness was ≤ 50% for only a single MAG, and contamination was ≥ 10 for three (Figure 5B). Of the species-classifiable MAGs, contamination was at maximum 5, but some had high CSS, up to 1.

Recall that only a small fraction of contigs was larger than 50 Kbp (Table 3). The average size of the contigs in the MAGs of samples 4 and 7 are 9.7 and 18.3 Kbp respectively (Figure 4C). The median NA50 of the classifiable MAGs are 8.5 and 35.3 Kbp respectively (Figure 5K), indicating that most of the assemble-able and mappable content of a MAG is concentrated within a small number of large contigs. The rest of the associated reference genome may be difficult to assemble and may have different sequence properties than the rest of the contigs. The use of BLAST or a taxonomic classifier (ex. Kraken2, MetaPhlan4, CAT [Wood et al., 2019, Blanco-Miguez et al., 2022, Cambuy et al., 2016]) to classify short contigs may be more appropriate, as they may be small fragments from low-abundant species too poorly covered by sequence content to bin into MAGs.

**Fig. 6.**
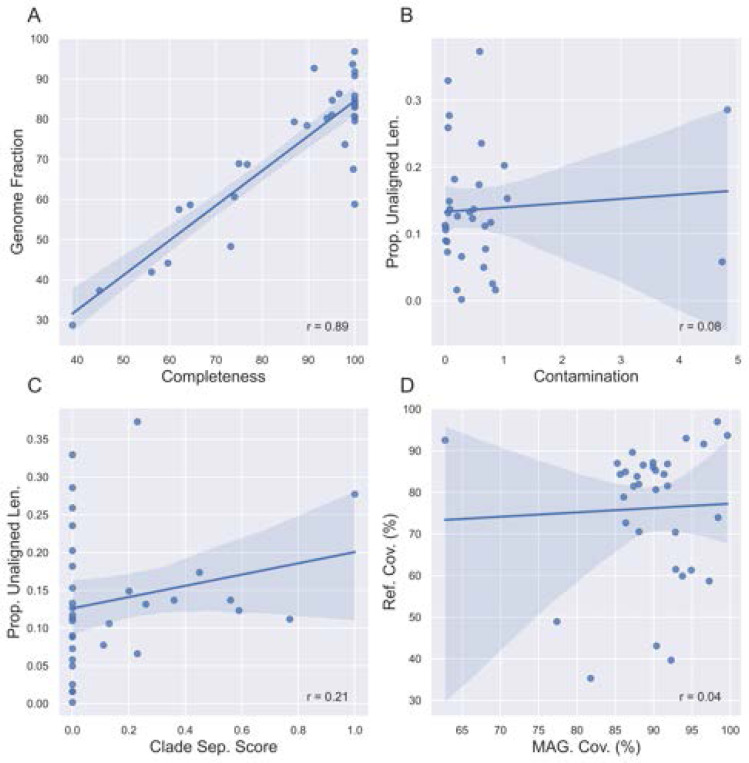
Reference-free metrics are sometimes correlated with reference-based metrics. (A) Completeness is highly correlated with QUAST-estimated genome fraction (the fraction of the reference genome that the MAG aligned to). (B, C) Contamination and clade separation score (CSS) do not correlate with the proportion of unaligned sequence material. (D) A larger proportion of the MAG aligns to the reference genome it is classified as, compared to the reference genome aligning to the MAG.

Completeness is highly correlated with QUAST-estimated genome fraction (the fraction of the reference genome that the MAG aligned to) (Figure 6A). However, contamination and CSS do not correlate with the proportion of unaligned sequence material (Figure 6B, C). Though both CheckM2 and gunc use a MAG’s protein content to estimate contamination, they differ in terms of application and thus interpretation. CheckM2 uses a neural network trained from almost protein-annotated 5,000 RefSeq bacterial genomes [Chklovski et al., 2023]. gunc classifies the candidate proteins and measures the taxonomic entropy within a contig and the MAG overall [Orakov et al., 2021]. If a MAG is high in CheckM2-estimated contamination but low in gunc-estimated CSS, then the MAG’s extraneous sequence content likely belongs to the same or a related taxon but still contains extra protein. The MAG may be a chimera of multiple highly related species or strains. Conversely, if a MAG is low in CheckM2-estimated contamination but high in gunc-estimated CSS, the extraneous sequence content may be non-redundant proteins from more distantly related taxa (ex. different genera). Both metrics only consider the over- or under-abundance of protein content. If there are contaminant non-protein-coding sequences, or sufficient synonymous mutation-based variation, that unalignable sequence material would not be considered by either metric.

Consistently, a larger proportion of the MAG is alignable to the reference genome it is classified as, rather than the converse (Figure 6D). Much of the reference genome material is missing in the MAG, which is corroborated by the median sizes of the MAGs-3 Mbp, which is small for a bacterial genome (Figure 4C, Figure 5H) [diCenzo and Finan, 2017]. Since the median average nucleotide identity between a MAG and the reference genome of its classified species (if available) was 97.34 (Figure 5E), the inferred MAGs are likely a specific substrain of the associated species. For example, the most abundant MAG in sample 4 was classified as *Cutibacterium acnes* and had an average coverage of 55.1X. 93.7% of the species reference genome was present in the MAG, and there were 573 positions with alternate alleles relative to the reference.

#### Comparing Minimum Contig Size Thresholds on MAG Quality

To determine if the minimum contig size threshold had appreciable impacts on MAG recovery and quality, we also binned Samples 3 and 7 (the smallest and largest short-read sequencing datasets, respectively) with a minimum size threshold of 1000 bp instead of 2500 bp. The results were highly dataset-dependent, and displayed no clear trends (Supplementary Materials). For maximally robust MAGs, it may be beneficial to bin assemblies with multiple minimum contig size thresholds, and then use an ensemble or dereplication tool like DAS Tool or dRep to find the most robust contig clusterings.

As expected, the sample 7 MAGs had lower completeness and contamination when binned with a 2500 bp threshold than 1000 bp. Conversely, the sample 3 MAGs were higher in both quality metrics with a threshold of 2500 bp (Supp. Table 4). If the species-classified MAGs are considered alone, the average genome fraction (though not completeness) is higher with the 2500 bp cutoff, despite having the same average MAG sizes (Supp. Figure 2H, I). One potential explanation The MAGs assembled with the 1000 bp threshold also had proportionally more unaligned contigs and bases (Supp. Figure 2N, O). In terms of recovered species, the same taxa were recovered in both 1000 and 2500 bp binning attempts of sample 3, and the MAGs classified as *D. sp003523545* and *M. sp001878835* were larger with the 2500 bp threshold (Supp. Table 3). Conversely, the 2500 bp attempt for Sample 7 did not recover *Chimaeribacter coloradensis* specifically, only a MAG that was classified as Chimaeribacter at the genus level (Supp. Table 3). The 1000 bp attempt recovered both the species C. coloradensis as well as a separate genus-only MAG, although the MAG was larger than the reference genome size (5.6 Mbp vs. 4.7 Mbp respectively), and the contamination and CSS were high (22.66, 0.52). The Pseudomonas B.-classified MAG in the 2500 bp attempt was larger than the P.luteola-classified MAG in the 1000 bp atempt, but the completeness and contamination/clade separation scores were low and high respectively. Thus, the MAG inferred with the 2500 bp size threshold is likely a chimera of several Pseudomonas species, as noted previously.

### Short-read Taxonomic Classification

The short-read taxonomic classification Jupyter notebook semi-automatically compares the 30 technical replicates (10 samples x 3 classifiers) using 2 alpha diversity metrics (taxonomic richness (i.e.: number of taxa and Shannon entropy) and 2 beta diversity metrics (Bray-Curtis dissimilarity and Jaccard distance) at different taxonomic ranks: phylum, class, order, family, genus, and species.

**Fig. 7.**
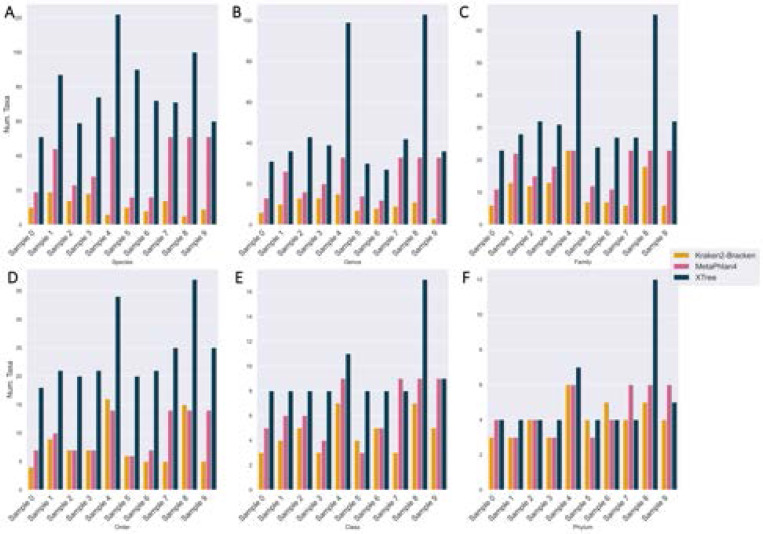
Each classifier detects different numbers of taxa across all ranks. (A) Species. (B) Genus. (C) Family. (D) Order. (E) Class. (F) Phylum.

**Fig. 8.**
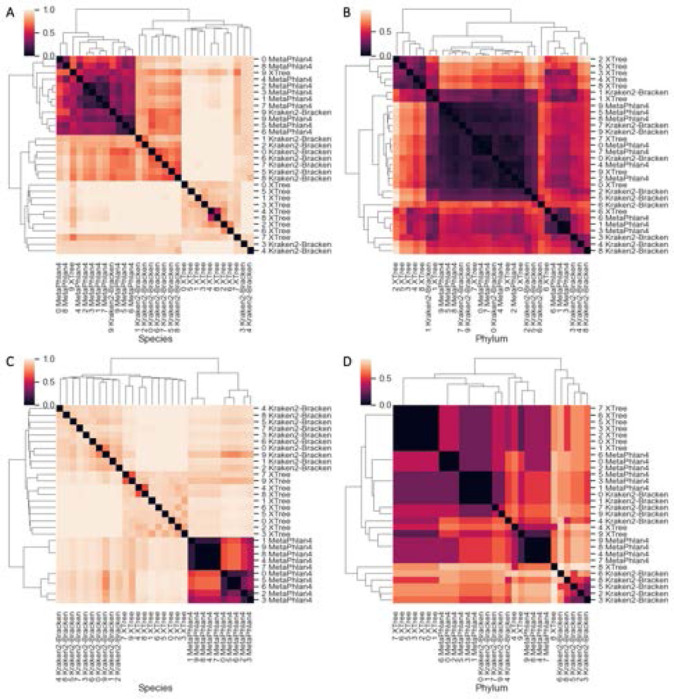
Taxonomic profiles are classifier, not sample-specific. Bray-Curtis and Jaccard distances between each replicate’s species (A, C) and phylum (C, D) profiles.

**Fig. 9.**
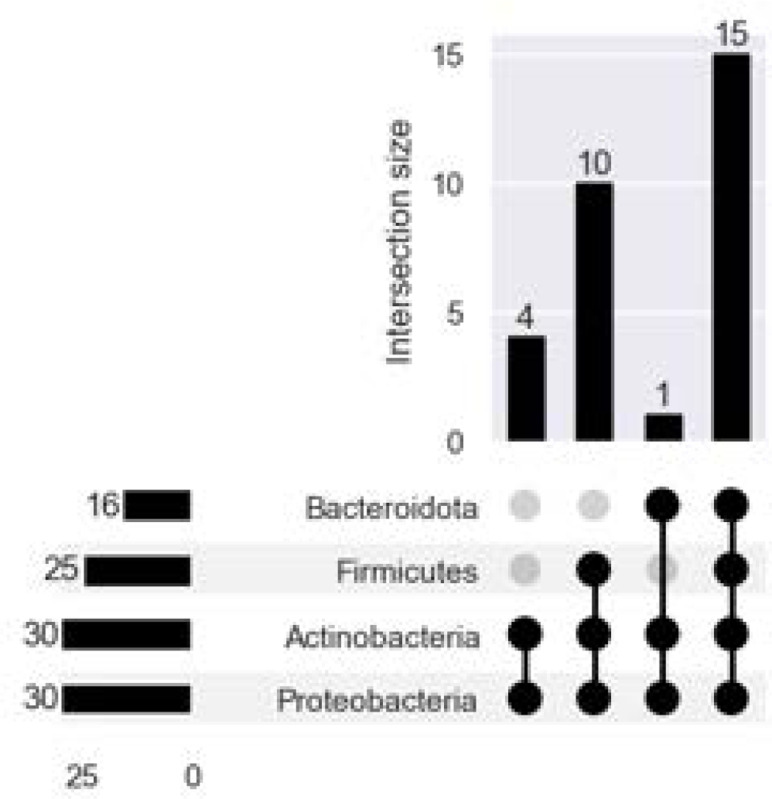
There are four phyla present in a majority but not all of the 10 samples: Proteobacteria, Actinobacteria, Firmicutes, and Bacteroidota.

Across all taxonomic ranks, XTree tended to discover the largest number of taxa (Figure 7). When comparing shared taxa across all 30 technical replicates, both the Bray-Curtis and Jaccard distances between each replicate’s species profile indicates that there is almost total assortment of species by classifier (Figure 8A, C). 630 unique species were discovered across three classifiers, with the vast majority (599/630, 95.1%) found by a single classifier. Only 6/630 (0.95%) and 25/630 (3.97%) species were found by all 3 and 2 of the 3 classifiers respectively, and the estimated relative abundance of the universally discovered species (averaged across all samples they were discovered in) varies (Table 5). At the higher rank of phylum, there is less distinctive classifier-specific assortment, but the taxonomic profiles of the same sample generated by different classifiers tend not to cluster together (Figure 8B, D). There are 4 phyla are present in most classifier-sample replicates: Proteobacteria, Actinobacteria, Firmicutes, Bacteroidota (Figure 9), which were previously noted as the most prevalent 4 phyla by the original MetaSUB publication [Danko et al., 2021]. The relative abundance accounted for by all these phyla (per sample) are 0.912 (±0.155) ranging from [0.443, 1].

We conducted a small case study on the species discovered by the 3 classifiers in sample 7, and found that each classifier’s associated database contains some but not all the species found in the others (Supplementary Materials). Unless custom databases are built from the same reference genome source, or multiple tools are used, it is difficult to compare classification strategies.

#### Comparing Short-Read Discovered and MAG Species

The number of unique species discovered across all three short-read taxonomic classifiers far outnumbers the number of MAGs in each sample (Figure 7A, Table 4). If we consider a more stringent filter and only consider species that have been discovered by at least 2 classifiers with a minimum relative abundance of at least 0.01, then the number of ‘verified’ species is much closer to the number of MAGs. However, the species discovered in by the short-read classifiers generally do not match the species classified by the MAGs (Table 6). The exception is sample 4, where every species-classifiable MAG (3 of 4 MAGs in total) was also discovered in the short-read shortlist. The only MAG that was not found in the list was classified only as a member of genus Janibacter, which was not found by at least 2 of the short-read classifiers. Of the species in Table 6, there 17 ‘verified’ short-read and 17 MAG species that do not appear in the list of 75 most abundant taxa (i.e.: the ‘core’ and part of the ‘subcore’ global urban microbiome) in [Danko et al., 2021]. The most abundant bacterial species from the original study- *Cutibacterium acnes*- appears in the 3/10 samples (Table 6) [Danko et al., 2021].

When considered in concert with the short-read assembly results, the largest samples by dataset and assembly size (in terms of the number of reads and/or bases)- 4, 7, 8, and 9- are also the samples with the largest number of ‘verified’ species, indicating some positive correlation between absolute amounts of overall sequence content and captured taxonomic diversity.

While there are likely more species per sample than binning algorithms are inferring, it is likely that the undiscovered species were not complete or stable enough for DAS Tool to confidently associate sets of contigs with each other.

### Virus and Phage Inference

A small fraction of the total number of contigs are flagged by the virus/phage-inference algorithms as potentially containing viral or phage sequence matter (Supp. Table 7). However, of those contigs, only around 10% of the flagged contigs are classifiable at the species i.e.: align to a virus or phage reference genome in RefSeq.

The largest number of flagged contigs in sample 7, which had the largest number of reads and assembly in terms of bases (Supp. Table 7). CheckV quality does not map well to RefSeq classifiability; only 17/26, 65.4% of CheckV-assessed complete viruses were species-classifiable. At best, the average CheckV-assessed completeness was on average 38.2% in sample 3, which contained 2044 algorithm flagged contigs (the second-most, next to sample 7) (Supp. Table 8).

**Fig. 10.**
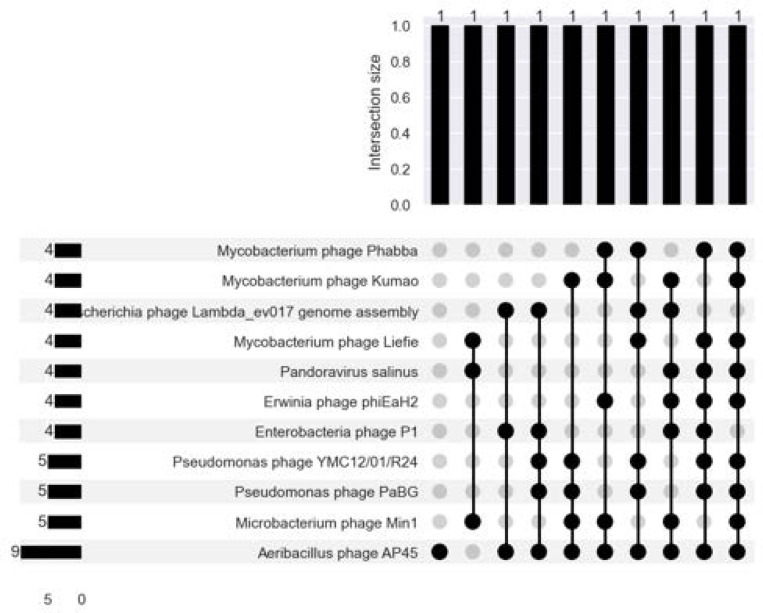
There is more overlap between the virus and/or phage profiles of different samples than there are bacteria.

There are 332 unique species-classifiable viruses, with the largest number in the sample with the largest number of reads, assembled bases, flagged contigs, and Blast-aligned contigs. Of these, 259/332 (78.0%) are unique to a single sample. The same virus or phage species was typically discovered multiple times in the same sample (Table 7). Taking the low completeness per contig into account, each contig could either be fragments of the same isolate, or there could be viral/phage strain diversity in the sample despite the low (‘verified’) bacterial diversity. 11/332 (3.3%) of species were found in 4+ samples (Figure 10). Of these, 10/11 (90.9%) aligned to bacteriophage reference genomes.

#### Comparing Virus-Phage and MAG Classifications

To maintain consistency from the short-read taxonomy section, we selected sample 4 for a case study. When we cross-referenced algorithm-flagged contigs with contig assignments to MAGs, we found that 20/44 (45.5%) candidate virus/phage contigs are found in MAGs. Only 1/44 (2.3%) contigs is labeled as a provirus by CheckV- Bixzunavirus I3. The contig is associated with a MAG, but MAG was classified as *Micrococcus luteus* and is not of the genus Mycobacterium, which is the phages’ known host [Reddy and Gopinathan, 1987].

As with the above example, most of the phage-MAG associations are not within the narrow host range of phages. Most phages can only infect several strains of bacteria from the same species, though there are documented cases of phages infecting bacteria from different genera [Ross et al., 2016]. Of the non-phage contigs (8/44, 18.2%), none are associated with MAGs except 3/4 Pandoraviruses, which are oddly associated with Micrococcus luteus.

### Gene Cataloguing

There are 407, 098 individual gene annotations found across all contigs across all samples with an average of 3.974 ±28.98 (range: [1, 3642]). The median number of genes annotated per contig is 1, which agrees with the shortness of most contigs (per-sample averages are 262 – 930 bp). The vast majority of contigs are extremely short- >99.5% are <2500 bp.

**Fig. 11.**
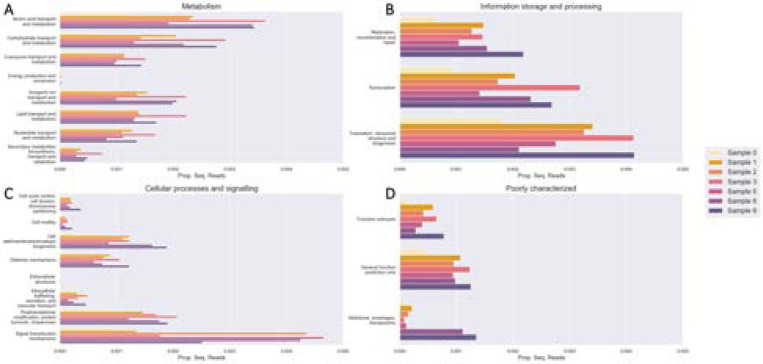
(A) Metabolism, (B) information and storage, (C) cellular process and signaling, and (D) poorly characterized clusters of orthologous genes (COGs) annotated in each sample. The x-axis refers to the proportion of sequencing reads aligned to genes from a COG.

Most genes cannot be assigned to a cluster of orthologous genes (COG), which are families of orthologous protein-coding genes (Supp. Table 9). Generally, the larger the dataset and/or assembly, the more COG representatives are found in the assembly. Samples 4, 7, and 8 do not have gene annotations because the associated Bakta jobs did not complete. Most genes, if they are assigned to a COG, belong to the metabolism category (Figure 11A). However, the COG with the largest number of genes is that of translation and ribosomal structure (Figure 11C). There were very few genes annotated in energy production and conversion, extracellular structures, and cytoskeleton COGs (Figure 11). It is unknown whether urban microbiome assemblies simply contain very few genes of those functions, or are they difficult to assemble or annotate (ex. not conserved enough or difficult to find via HMM). There were not many cell motility-annotated genes either, which is interesting given one would expect to find many free-living species in the urban milieu.

### Computational Resources Used

If executed as an end-to-end workflow, all modules in our pipeline can be used to efficiently process sequencing data on a high-performance computing (HPC) cluster. The workflow was designed to optimize resource utilization and minimize processing time (Table 8). Our configuration allowed us to process large-scale metagenomic datasets efficiently, enabling comprehensive analysis of microbial communities across multiple samples. The modular nature of our workflow allows for scalability and adaptability to different computational environments, from local workstations to cloud-based infrastructures.

## Discussion

Rapid advances in both wet- and dry-lab metagenomics techniques have facilitated comprehensive studies of microbial communities’ diversity and functional capabilities. However, computational analysis of large metagenomic data presents several challenges, including the need to extract accurate taxonomic and functional insights from datasets varying in size, species complexity, and diversity. To address these challenges, we developed CAMP, a modular metagenomics analysis system that is designed to enable integrated multi-analysis step data exploration and facilitate automated tool benchmarking and comparisons. The architecture of CAMP allows for the execution of several tools as discrete single-input to single-output units, suitable for highly study-specific downstream steps (e.g., from raw sequencing reads to quality-filtered sequencing reads), or for orchestrating dozens of tools in full end-to-end or multi-pronged workflows (e.g., from raw sequencing reads to short-read classification as well as metagenome-assembled genome (MAG) reconstruction). Importantly, CAMP offers an easy-to-install procedure and is compatible with various computing systems. The modular architecture of the CAMP makes module customization and future expansions straightforward for both core and external developers.

The application of CAMP to a 10-sample urban microbiome dataset demonstrates the utility of having automated data visualization for integrated analyses across every step of a processing workflow. By integrating results from the MAG binning, short-read taxonomic classification, and virus/phage inference modules, we were able to compare and contrast the taxonomic diversity inferred by various methods, as well as intermediate results from short-read pre-processing assembly to properly contextualize them.

Thanks to the standardized module structure, new Snakemake rules can easily be added to expand existing modules, and new study-specific modules can be created using the template. There are many modules currently under active development, including Oxford Nanopore long-read preprocessing, long-read taxonomic profiling, and decontamination. Future extensions for new avenues of investigation that have been demonstrated with existing one-click pipelines include long-read and hybrid short-long assembly, multi-sample coassembly, variant and strain analysis, metatranscriptomics, and functional and pathway annotation. Additionally, for users with root access, we plan to offer containerized steps using Singularity to enhance reproducibility and interoperability, as well as cloud support.

## Conclusion

CAMP is ideal for researchers and laboratories looking to set up a standardized analysis workflow for a large-scale metagenomics analysis with multiple hypotheses and/or research topics that need to be addressed with many data types. Singular CAMP modules are also suitable for smaller-scale projects that require deep data exploration. The standardized output dataframes can also serve as inputs for customized machine learning ingestion.

## Supplementary Material

Supplement 1

## Figures and Tables

**Table 1.** Each feature in the module system was designed to maximize scalability (in terms of dataset size as well as system distribution), portability (i.e.: compatibility with existing analysis environments), ease of use, and analysis transparency and reproducibility.

Feature	How does it facilitate best-practices?
1) Standardized module template	• **Ease of use**: Every single module in the system has the same structure as described in Box 9, which makes it extremely easy to i) learn how to use existing modules, ii) customize existing modules with new tools and rules, and iii) make completely new modules. – The module template is a Cookiecutter [Cookiecutter, 2022] that can be used to start new modules from scratch in a few minutes. • **Scalability**: Thanks to a fully automated Cookiecutter-based template, the entire system is extremely flexible and can easily integrate developments in the metagenomics field. New modules for custom purposes easily be set up with the template and connected to existing modules in the system. For example, a hybrid short-long read assembly module can use as inputs the output samples.csv files from short- and long-read preprocessing.
2) Each module is a self-contained analysis unit	• Soft pauses are built into the end of every module so that the user can visually inspect the intermediate output and apply additional analysis steps that may not have been apparent at the outset. • **Portability**: Each module can be downloaded and set up individually. For example, if a user just wants to do short-read taxonomic classification, they only need the short-read preprocessing and classification modules and do not need to set up anything MAG-related.
3) Click-based Python command-line interface	• Snakemake’s API is unwieldly for coordinating multiple command-line parameters and computational resources, not to mention restarting and rerunning existing jobs. The intuitive Python-based command-line wrapper internally communicates with Snakemake’s API and allows users to interact with an easily operated interface. • **Ease of use**: The interface eliminates the need to write long, unwieldy, error-prone Snakemake commands.
4) Standardized input-output file format	• The input and output of every module is always in a standardized samples.csv format that encodes the locations of input or output data for each sample in the dataset. • **Scalability**: This simple file format is the glue that allows modules to be joined together into whatever complexity of workflow the user needs.
5) Standardized working directory structures	• **Ease of use**: Similar to the standardized module directory, once the user is familiar with navigating the intermediate and output files of one module, all others are equally navigable. • **Transparency and reproducibility**: Intermediate and output reports as well as job logs are located in predictable and intuitive directory structures. If jobs fail, finding the error, whether it is due to computational under-resourcing or incorrectly set parameters, is straightforward.
6) Summary statistics of module outputs for accessible first-pass interpretations	• **Scalability**: Large datasets can be difficult to analyze without intuitive summaries. For example, the short-read taxonomic classification module comes with the taxa discovered by each of three classification algorithms as well as their estimated relative abundances in standardized CSVs for easy comparisons. • **Portability, transparency, and reproducibility**: By providing summaries at the end of each module, users can document every step of their analysis parsimoniously without having to keep every single large intermediate file.
7) Jupyter notebook-based semi-automated visualization of summary statistics	• **Ease of use and scalability**: While summary statistics are useful for documentation, visualizations are a more intuitive way to understand dataset outputs, especially large datasets with many samples that need to be compared.
8) Module-independent files for parameter and computational resource settings	• **Scalability**: While a default set of parameter and resource values are provided with the sample config YAML, users can set different parameter and resource profiles depending their data. • **Portability, transparency, and reproducibility**: To share the parameter and resource settings used in their analyses, users need only share these two text files. • **Ease of use**: Users only have to refer to these two text files to adjust parameters and resources without creating complex data structures on the command line.
9) Included test dataset and sample outputs from test dataset	• **Portability**: The user can verify proper module setup, as well as visually inspect the proper intermediate and output file formats and work directory to gain mechanistic familiarity the purpose of each step in the module.
10) Conda-based environment setup with integrated YAMLs	• **Scalability**: Instead of re-installing existing Conda environments into every analysis working directory, Conda environments are built directly into the module directory only once. • **Portability and ease of use**: Complex conflict-free environments are deployed along with the module workflow code, and can be easily set up using standard Conda commands. • **Transparency and reproducibility**: Environments are standardized and auto-documented for version consistency.
11) Slurm job scheduler integration	• **Scalability**: Allowing modules to be run with job scheduler assistance helps users with access to HPCs to parallelize running rules within the module.

**Table 2.** Ten randomly chosen urban microbiome samples from the MetaSUB New York dataset along with metadata such as their dates and locations of origin.

Sample ID	Full Sample Name	Sampling Time	Train Line	Transit Station (Borough)	Swabbed Surface	Num. Read Pairs
0	haib17CEM5106_HCY5JCCXY_SL269539	Winter 2014	D	NA	Subway train seat	5933694
1	haib17CEM5106_HCV72CCXY_SL269726	Winter 2014	6	59 St. (Manhattan)	NA	2002519
2	haib17CEM5106_HCVMTCCXY_SL269540	Winter 2014	D	NA	Subway train seat	2864273
3	haib17CEM5106_HCV72CCXY_SL269724	Winter 2014	6	Grand Central (Manhattan)	NA	1814764
4	haib17DB4959_H3MGVCCXY_SL259896	June 2017 (City Sampling Day)	NA	Van Siclen Av. (Brooklyn)	Transit station bench	8285289
5	haib17CEM5106_HCV72CCXY_SL269741	Winter 2014	6	NA	Subway train ceiling handrail	3864641
6	haib17CEM5106_HCY5JCCXY_SL269601	Winter 2014	NA	36th St. (Queens)	Transit station turnstile	3760124
7	haib17CEM4890_H2NYMCCXY_SL254808	June 2016 (City Sampling Day)	NA	Halsey St. (Brooklyn)	Transit station turnstile	23076691
8	haib17DB4959_H3MGVCCXY_SL259845	June 2017 (City Sampling Day)	NA	Flushing-Main St. (Queens)	Transit station escalator handle	5349073
9	haib17CEM4890_HMCMJCCXY_SL335779	June 2016 (City Sampling Day)	NA	33rd St. (Manhattan)	Transit station kiosk	4834819

**Table 3.** Number and proportion of contigs and bases at each size threshold

Sample	Min. Contig Size	Num. Contigs (Prop.)	Num. Bases (Drop.)
4	0 bp	1636542 (1.000)	447963811 (1.000)
4	2,500 bp	1872 (0.001)	10832446 (0.024)
4	50,000 bp	12 (0.000)	1000212 (0.002)
4	100,000 bp	3 (0.000)	342030 (0.001)
7	0 bp	1217385 (1.000)	361500456 (1.000)
7	2,500 bp	6605 (0.005)	53796944 (0.149)
7	50,000 bp	94 (0.000)	11802085 (0.033)
7	100,000 bp	41 (0.000)	8342312 (0.023)

**Table 4.** The number of MAGs inferred by the 6 binning algorithms and ensemble-aggregated by DAS Tool, as well as the number of MAGs that can be classified by GTDB-Tk

Sample	Num. DAS Tool MAGs	Num. Classifiable MAGs	Num. Species-Classifiable MAGs
0	4	4	3 (75%)
1	4	4	4 (100%)
2	8	8	4 (50%)
3	6	6	5 (83.3%)
4	4	4	3 (75%)
5	7	7	5 (71.4%)
6	5	5	5 (100%)
7	3	3	1 (33.3%)
8	2	2	2 (100%)
9	1	1	1 (100%)

**Table 5.** Comparison of species abundance across different tools

Species	Kraken2/Bracken	MetaPhlAn4	Xtree

Acinetobacter lwoffii	0.039822	0.382999	0.019473
Acinetobacter radioresistens	0.004897	0.052929	0.009076
Acinetobacter schindleri	0.181852	0.159433	0.018861
Acinetobacter variabilis	0.006946	0.069463	0.008656
Kocuria rosea	0.001764	0.005049	0.000509
Mixta calida	0.010038	0.101190	0.009403

**Table 6.** Most ‘verified’ (i.e.: discovered by at least 2 classifiers, minimum relative abundance of 0.01) short-read species do not overlap with MAG species, and vice versa.

Sample	Short-Read Species	MAG Species
Sample 0	Acinetobacter schindleri, Acinetobacter lwoffii, Rothia terrae	Agrobacterium pusense, Corynebacterium variabile, Aerococcus sp002440555
Sample 1	Acinetobacter schindleri, Acinetobacter lwoffii, Desemzia incerta, Flavimobilis marinus	Stenotrophomonas maltophilia N, Psychrobacter sp001652315, Brevibacterium iodinum, Timonella senegalensis
Sample 2	Acinetobacter schindleri, Acinetobacter lwoffii, Kocuria rosea, Staphylococcus hominis, Staphylococcus epidermidis	Micrococcus luteus, Epilithonimonas sp002391865, Staphylococcus aureus, Kocuria salsicia
Sample 3	Acinetobacter schindleri, Acinetobacter lwoffii, Arsenicicoccus bolidensis, Micrococcus luteus	Dysgonomonas sp003523545, Enterococcus faecalis, Leuconostoc mesenteroides, Pseudomonas A stutzeri, Microbacterium sp001878835
Sample 4	Acinetobacter schindleri, Kocuria rosea, Cutibacterium acnes, Arsenicicoccus bolidensis, Micrococcus luteus, Sphingomonas aerolata, Kocuria palustris, Deinococcus radiopugnans, Corynebacterium aurimucosum, Psychrobacter faecalis	Deinococcus radiopugnans, Cutibacterium acnes, Micrococcus luteus
Sample 5	Acinetobacter schindleri, Acinetobacter lwoffii	Microbacterium foliorum A, Corynebacterium ammoniagenes, Carnobacterium A jeotgali, Microbacterium paraoxydans, Rhodococcus erythropolis D
Sample 6	Acinetobacter schindleri, Staphylococcus hominis	Microbacterium saccharophilum, Epilithonimonas sp002391865, Lactococcus lactis, Pseudomonas E rhodesiae, Dermacoccus nishinomiyaensis
Sample 7	Acinetobacter schindleri, Acinetobacter lwoffii, Acinetobacter variabilis, Acinetobacter radioresistens, Cutibacterium acnes, Acinetobacter pittii, Leuconostoc lactis, Pantoea septica, Psychrobacter faecalis	Leuconostoc lactis
Sample 8	Acinetobacter schindleri, Kocuria rosea, Cutibacterium acnes, Arsenicicoccus bolidensis, Micrococcus luteus, Sphingomonas aerolata, Piscicoccus intestinalis, Kocuria palustris, Deinococcus radiopugnans, Deinococcus reticulitermitis, Corynebacterium aurimucosum	Cutibacterium acnes, Actinomyces oris A
Sample 9	Acinetobacter schindleri, Acinetobacter lwoffii, Acinetobacter variabilis, Acinetobacter radioresistens, Mixta calida, Kocuria rosea, Weissella confusa, Cutibacterium acnes, Rothia terrae, Staphylococcus hominis, Acinetobacter johnsonii	Mixta calida

**Table 7.** Each virus or phage species discovered in a sample is represented by on average > 1 contig in that sample.

Sample	Num. Viruses/Phages	Avg. Num. Contigs

Sample 0	15	1.40
Sample 1	45	1.71
Sample 2	60	1.82
Sample 3	61	3.64
Sample 4	36	1.22
Sample 5	16	1.63
Sample 6	37	2.95
Sample 7	130	3.32
Sample 8	8	1.38
Sample 9	37	2.19

**Table 8.** Time required to process all 10 samples with 64 CPUs available to the Snakemake command.

Module	Time (dd:hh:mm:ss)

Short-read preprocessing	00:00:34:03
Short-read assembly	00:08:14:05
MAG binning	00:01:45:12
MAG quality-checking	00:01:56:50
Short-read taxonomy	05:22:35:42
Virus-phage inference	03:17:31:18
Gene cataloguing	03:15:08:22

## Data Availability

The raw sequencing sequencing data can be found under the SRA accession ID PRJNA732392, as well as the GeoSeeq repository.

## Box 1: Standardized module architecture for system-wide navigability and extendability.

workflow/: Contains all of the module’s functional code and scripts from external tools that are under an open-source license.

module_slug.py: The Click-based command-line interface that wraps a Snakemake-based workflow.

Snakefile: Workflow that itself wraps Snakemake rules, which call external algorithms or internal functions (utils.py) to process data.

utils.py: Internal functions such as dataset ingestion and working directory setup.

ext/: External scripts and smaller data files required by algorithms used in the workflow.

configs/: Contains all settings files.

parameter.yaml and resource.yaml: Instead of relying on Snakemake’s dictionary-based system of specifying parameters and resources on the command line,algorithm parameters and computational resource allocations have been unified into single YAML files. Users can:

i) use the default YAMLs provided, ii) modify the values in the YAMLs according to their dataset properties, or iii) make multiple copies of the YAMLs to analyze datasets of different sizes and biological origins.

samples.csv: A standardized input file where each row corresponds to a sample. The first column is always the sample’s name, and every column thereafter contains paths to that sample’s data. conda/: Conda environment recipes. The main environment is always called module_slug.yaml and should be set up immediately after cloning the module to a local directory. All other Conda environments will be set up the first time the workflow is run into a directory called conda_envs/in the module’s top-level directory.

sbatch/: A Snakemake profile that enables the module’s rules to be run using the Slurm job scheduler.

test_data/: Test data to ensure proper module setup, and an example work directory to demonstrate its structure, and input, intermediate, and output file formats.

dataviz.ipynb: Guided and semi-automated data visualization of the module’s final output using Jupyter notebook. Users can use the built-in graphing functions as is or customize as needed.
