# Supplementary material for: CAMP: A modular metagenomics analysis system for integrated multi-step data exploration
Source: bioRxiv. 2024 Sep 14:2023.04.09.536171. Originally published 2023 Apr 9. Preprint. [Version 2] doi: 10.1101/2023.04.09.536171 (PMC10104186; doi:10.1101/2023.04.09.536171)
Supplement: Supplementary file 1 [file media-1.pdf]

METHODS

COMPARISON OF CAMP WORKFLOW AND METASUB METHODS

EXAMPLE 1: MAG QC

For example, the original MetaSUB study generated MAGs from each sample's assembly using a single binner and MIMAG-like completeness and contamination-based quality filters subsequently. To minimize the resource-heavy application of MAG classification, the MAGs were then clustered into operational taxonomic units (OTUs) using Mash and dnadiff. To classify the OTU, the representative MAG of an OTU was classified by calculating the MinHash distance to the closes reference genome in the RefSeq database (release 96 from November 2019) and the Integrated Gut Genomes (IGG) dataset (v1.0). In contrast, the CAMP MAG QC module is a purpose-agnostic workflow takes any set or sets of MAGs, characterizes them using reference-based and reference-free metrics, and summarizes the metrics with descriptive visualizations.

Using the MetaSUB example, users can generate a set of MAGs using the 7 binning algorithms built into the CAMP binning module, create and use custom MIMAG filters to dereplicate their MAGs according to the study's needs, and then plug that set of dereplicated MAGs into the CAMP MAG QC module for assessment and comparison.

EXAMPLE 2: SHORT-READ TAXONOMIC CLASSIFICATION

The original MetaSUB study generated short-read taxonomic classifications using KrakenUniq and two thresholds based on the number of assigned reads and marker k-mer uniqueness. In contrast, the CAMP short-read taxonomic classification module was designed to be a sandbox for testing and using multiple classification algorithms. To approximate classification robustness as in the original study, we use a relative abundance filter and the intersection of taxon discoveries from all three algorithms. As we have illustrated in the main text, database labeling differences means that counted taxa from different classification algorithms are largely non-comparable due to differences in database composition, reference genome labeling, and taxonomic organization (i.e.: different clusterings of reference genomes due to updates in the tree of life).

MAG QC

MODIFIED MIMAG STANDARDS

Quality	Metrics
High	CMComp > 90 and one of:<br>– CMCont ≤ 5 and 0.2 < CSS ≤ 0.45 or<br>– 5 < CMCont ≤ 10 and CSS ≤ 0.2
Medium	– CMComp > 90 and 5 < CMCont ≤ 10 and CSS ≤ 0.2 or<br>– 50 < CMComp ≤ 90 and CMCont ≤ 10 or<br>– 50 < CMComp ≤ 90 and CSS ≤ 0.45
Low	– CMComp > 50 and CMCont > 10 and CSS > 0.45 or<br>– CMComp ≤ 50

Supplementary Table 1: The modified minimum information about a metagenome-assembled genome (MIMAG) standards used to annotate CAMP MAGs. Abbreviations are as follows: CMComp- CheckM2-measured completeness, CMCont- CheckM2-measured contamination, CSS- gunc-measured clade contamination score.

RESULTS

Sample	Step	Num. Reads	Prop. Raw Reads	Num. Bases	Prop. Init. Bases	Mean Read Len. (bp)
4	Raw dataset	16,570,578	1.000	2,502,157,278	1.000	151
4	Low qual. removal	14,648,428	0.884	2,151,320,786	0.860	146.86
4	Adapter removal	14,648,416	0.884	2,143,774,115	0.857	146.35
4	Host read removal	13,141,634	0.793	1,922,660,878	0.768	146.3
4	Error correction	13,141,634	0.793	1,922,660,878	0.768	146.3
7	Raw dataset	37,524,480	0.813	5,416,628,326	0.777	144.35
7	Low qual. removal	37,524,480	0.813	5,415,978,268	0.777	144.33
7	Adapter removal	35,897,032	0.778	5,184,532,240	0.744	144.43
7	Host read removal	35,897,032	0.778	5,184,532,240	0.744	144.43
7	Error correction	35,897,032	0.778	5,184,532,240	0.744	144.43

Supplementary Table 2: The number and proportion of sequencing bases and reads retained in samples 4 and 7 at each short-read quality control step.

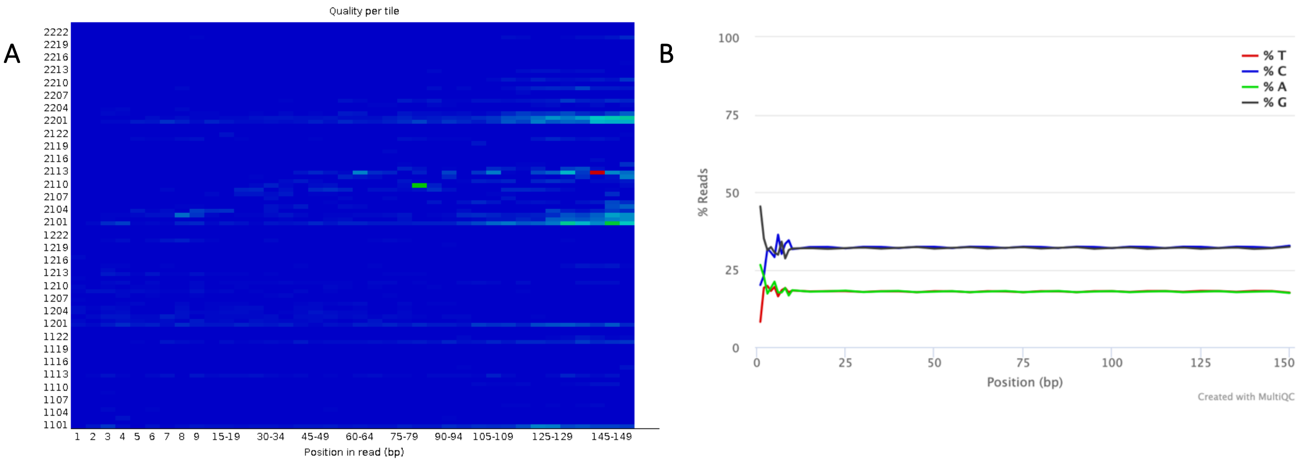

Supplementary Figure 1: Short-read quality-control plots. **A.** FastQC quality-per-tile heatmap of the reverse FastQ of sample 8. **B.** MultiQC per-base ACGT content plot of sample 4.

Sample	Largest Contig (bp)	Mean Contig Size (bp)	N50 (bp)
0	533,444	562.18	870
1	612,951	470.42	622
2	671,701	929.89	3845
3	195,037	637.41	1099

4	135,592	273.73	262
5	894,936	926.91	3016
6	537,484	800.44	5674
7	576,451	296.95	267
8	266,304	262.32	255
9	339,502	268.01	255

Supplementary Table 3: Various assembly contiguity statistics, as measured by MetaQUAST.

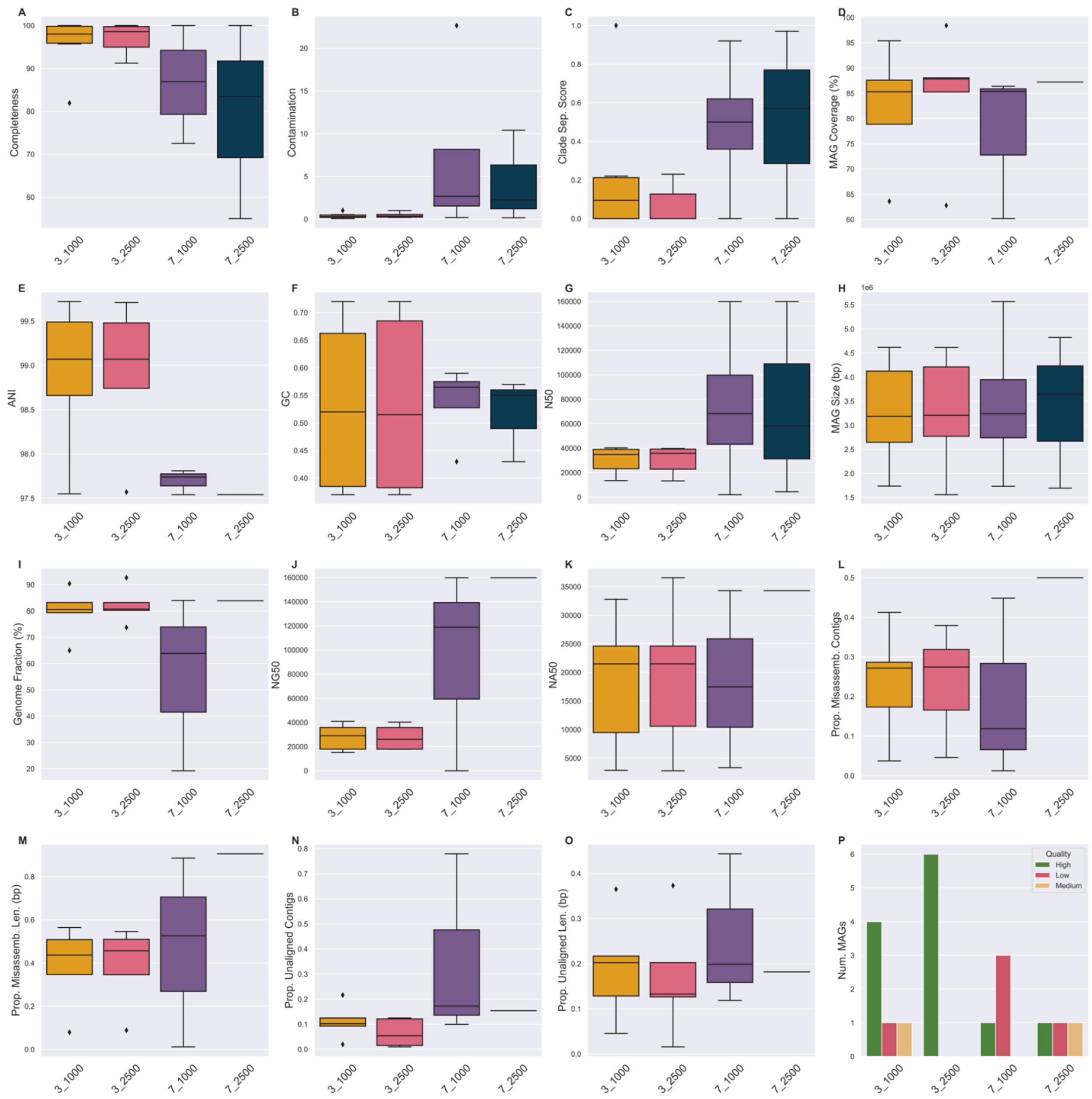

Supplementary Figure 2: Reference-based and reference-free quality metrics of MAGs inferred from samples 3 and 7 with minimum contig thresholds of 1000 vs. 2500 bp. Notation is `i_j`, where `i` is the sample number and `j` is the minimum contig size threshold (in bp).

Min. Contig Size (bp)	1000 bp	2500 bp
Sample 3		
Pseudomonas A stutzeri	S	S
Enterococcus faecalis	S	S
Microbacterium sp001878835	S	S
Leuconostoc mesenteroides	S	S
Dysgonomonas sp003523545	S	S
Mobilicoccus	G	G
Sample 7		
Pseudomonas B luteola	S	G
Leuconostoc lactis	S	S
Chimaeribacter	G	G
Chimaeribacter coloradensis	S	NA

Supplementary Table 4: Taxonomic classifications of MAGs recovered in samples 3 and 7 with minimum contig thresholds of 1000 vs. 2500 bp. 'S' indicates that a MAG corresponding to the species was identified by GTDB-Tk, 'G' indicates that a MAG corresponding to the genus was identified by GTDB-Tk, and 'NA' indicates that a MAG corresponding to that taxonomic lineage was not found (or closer to another taxon.

To investigate the cause behind the classifier specificity, we explored the reference genome database content of the selected classifiers. We used one of the largest datasets- sample 7 (largest number of reads, second-largest number of species discovered overall- 126) as a case study. We found that if the default databases are used for classification, 117/126 (92.9%) species discovered were classifier-specific. For each of the species (Supp. Table 5) and genera (Supp. Table 6) discovered by one classifier only, we searched each of the other classifiers' databases to determine if it was a presence issue (i.e.: database does not contain the species or genus) or a discovery issue (i.e.: species or genus was present in the database but the classifier failed to detect it). Most Kraken2-Bracken-only genera and species are not database-specific i.e.: both MetaPhlan4 and XTree databases contained 3/4 of the 'missing' genera at at least 8/13 of the 'missing' species. Conversely, all of MetaPhlan4- and XTree-only genera and species were not found in the Kraken2-Bracken database. Furthermore, out of the 62 XTree-only species, 62.9% of them were also not found in the XTree database.

		Database		
		Kraken2-Bracken	MetaPhlan4	XTree
Classifier	Kraken2-Bracken	-	5/13 (0.385)	7/13 (0.538)
	MetaPhlan4	42/42 (1.0)	-	17/42 (0.405)
	XTree	62/62 (1.0)	39/62 (0.629)	-

Supplementary Table 5: The number of species discovered by a single classifier (rows) that were missing in the reference databases of another classifier (columns). For example, 5/13 (38.5%) of the species discovered only by Kraken2-Bracken were missing in the MetaPhlan4 database. Likewise, 42/42 species discovered only by MetaPhlan4 were missing in the Kraken2-Bracken database.

		Database		
		Kraken2-Bracken	MetaPhlan4	XTree
Classifier	Kraken2-Bracken	-	1/4 (0.25)	1/4 (0.25)
	MetaPhlan4	26/26 (1.0)	-	3/26 (0.115)

	XTree	18/18 (1.0)	6/18 (0.33)	-

Supplementary Table 6: The number of genera discovered by a single classifier (rows) that were missing in the reference databases of another classifier (columns). For example, 1/4 (25%) of the genera discovered only by Kraken2-Bracken were missing in the MetaPhlan4 database. Likewise, 26/26 genera discovered only by MetaPhlan4 were missing in the Kraken2-Bracken database.

Sample	Tot. Num. Contigs	Num. Algorithm-Flagged	Num. BLAST-Aligned
Sample 0	70155	270	21
Sample 1	161405	1647	77
Sample 2	58395	1147	109
Sample 3	111732	2044	222
Sample 4	1636542	1841	44
Sample 5	61041	858	26
Sample 6	40518	566	109
Sample 7	1217385	3406	431
Sample 8	1138883	352	11
Sample 9	522136	778	81

Supplementary Table 7: The total number of contigs, the number of contigs flagged by ViralVerify, VIBRANT, VirSorter, or VirFinder as containing putative viral or phage genetic content, and the number of flagged contigs that were alignable by BLAST against a viral reference genome in the RefSeq database.

Sample	Num. Viruses/Phages	Avg. Num. Contigs
Sample 0	15	1.4
Sample 1	45	1.711
Sample 2	60	1.817
Sample 3	61	3.639
Sample 4	36	1.222
Sample 5	16	1.625
Sample 6	37	2.946
Sample 7	130	3.315
Sample 8	8	1.375
Sample 9	37	2.189

Supplementary Table 8: The number of virus/phage reference genomes found per sample by BLAST-ing contigs, as well as the average number of contigs found per reference genome.

COG category?	Num. candidate genes found	Num. unique candidate genes
Y	23132 (0.057)	2553 (0.104)
N	379159 (0.931)	21893 (0.896)

Supplementary Table 9: The number of candidate genes that were assignable to a cluster of orthologous genes (COG) category (proportion relative to the total number of genes in brackets), as well as the number of unique candidates (proportion relative to the total number of COG-annotated genes in brackets).

References

1. Chen, S., Zhou, Y., Chen, Y., & Gu, J. (2018). Fastp: An ultra-fast all-in-one FASTQ preprocessor. Bioinformatics, 34(17), i884–i890.
2. Bolger, A., Lohse, M., & Usadel, B. (2014). Trimmomatic: A flexible trimmer for Illumina sequence data. Bioinformatics, 30(15), 2114–2120.
3. Langmead, B., & Salzberg, S. (2012). Fast gapped-read alignment with bowtie 2. Nature Methods, 9(4), 357–359.
4. Li, H., Handsaker, B., Wysoker, A., Fennell, T., Ruan, J., Homer, N., Marth, G., Abecasis, G., & Durbin, R. (2009). The sequence alignment/map format and SAMtools. Bioinformatics, 25(16), 2078–2079.
5. Nikolenko, S., Korobeynikov, A., & Alekseyev, M. (2013). Bayeshammer: Bayesian clustering for error correction in single-cell sequencing. BMC Genomics, 14(Suppl 1).
6. Andrews S. (2010) A quality control tool for high throughput sequence data. http://www.bioinformatics.babraham.ac.uk/projects/fastqc/.
7. Ewels, P., Magnusson, M., Lundin, S., & Käller, M. (2016). MultiQC: Summarize analysis results for multiple tools and samples in a single report. Bioinformatics, 32(19), 3047–3048.
8. Nurk, S., Meleshko, D., Korobeynikov, A., & Pevzner, P. (2017). Metaspades: A new versatile metagenomic assembler. Genome Research, 27(5), 824–834.
9. Li, D., Liu, C.M., Luo, R., Sadakane, K., & Lam, T.W. (2015). Megahit: An ultra-fast single-node solution for large and complex metagenomics assembly via succinct de Bruijn graph. Bioinformatics, 31(10), 1674–1676.
10. Mikheenko, A., Saveliev, V., & Gurevich, A. (2015). MetaQUAST: evaluation of metagenome assemblies. Bioinformatics, 32(7), 1088–1090. https://doi.org/10.1093/bioinformatics/btv697
11. Kang, D., Li, F., Kirton, E., Thomas, A., Egan, R., An, H., & Wang, Z. (2019). MetaBAT 2: An adaptive binning algorithm for robust and efficient genome reconstruction from Metagenome Assemblies. PeerJ.
12. Alneberg, J., Bjarnason, B., Bruijn, I., Schirmer, M., Quick, J., Ijaz, U., Lahti, L., Loman, N., Andersson, A., Quince, C., & et al. (2014). Binning metagenomic contigs by coverage and composition. Nature Methods, 11(11), 1144–1146.
13. Pan, S., Zhao, X., & Coelho, L. P. (2023). SemiBin2: self-supervised contrastive learning leads to better MAGs for short- and long-read sequencing. Bioinformatics, 39(Supplement_1), i21–i29. https://doi.org/10.1093/bioinformatics/btad209
14. Nissen, J., Johansen, J., Allesøe, R., Sønderby, C., Armenteros, J., Grønbech, C., Jensen, L., Nielsen, H., Petersen, T., Winther, O., & et al. (2021). Improved metagenome binning and assembly using deep variational autoencoders. Nature Biotechnology, 39(5), 555–560.
15. Wu, Y.W., Simmons, B., & Singer, S. (2015). MaxBin 2.0: An automated binning algorithm to recover genomes from multiple metagenomic datasets. Bioinformatics, 32(4), 605–607.
16. Wang, Z., Huang, P., You, R., Sun, F., & Zhu, S. (2023). MetaBinner: a high-performance and stand-alone ensemble binning method to recover individual genomes from complex microbial communities. Genome Biology, 24(1). https://doi.org/10.1186/s13059-022-02832-6
17. Sieber, C., Probst, A., Sharrar, A., Thomas, B., Hess, M., Tringe, S., & Banfield, J. (2017). Recovery of genomes from metagenomes via a dereplication, aggregation, and scoring strategy. Nature Microbiology.
18. Chklovski, A., Parks, D. H., Woodcroft, B. J., & Tyson, G. W. (2023). CheckM2: a rapid, scalable and accurate tool for assessing microbial genome quality using machine learning. Nature Methods, 20(8), 1203–1212. https://doi.org/10.1038/s41592-023-01940-w

19. Orakov, A., Fullam, A., Coelho, L. P., Khedkar, S., Szklarczyk, D., Mende, D., Schmidt, T., & Bork, P. (2021). GUNC: detection of chimerism and contamination in prokaryotic genomes. Genome Biology, 22(1). https://doi.org/10.1186/s13059-021-02393-0
20. Chaumeil, P.A., Mussig, A., Hugenholtz, P., & Parks, D. (2019). GTDB-TK: A toolkit to classify genomes with the Genome Taxonomy Database. Bioinformatics.
21. Gurevich, A., Saveliev, V., Vyahhi, N., & Tesler, G. (2013). Quast: Quality Assessment Tool for Genome Assemblies. Bioinformatics, 29(8), 1072–1075.
22. Wood, D., Lu, J., & Langmead, B. (2019). Improved metagenomic analysis with Kraken 2. Genome Biology.
23. Lu, J., Breitwieser, F., Thielen, P., & Salzberg, S. (2017). Bracken: Estimating species abundance in metagenomics data. PeerJ Computer Science, 3.
24. Blanco-Miguez, A., Beghini, F., Cumbo, F., McIver, L., Thompson, K., Zolfo, M., Manghi, P., Dubois, L., Huang, K., Thomas, A., & et al. (2022). Extending and improving metagenomic taxonomic profiling with uncharacterized species with metaphlan 4. bioarXiv.
25. GabeAl. (2022). Gabeal/Utree: K-Mer searching with trees. https://github.com/knights-lab/UTree
26. Antipov, D., Raiko, M., Lapidus, A., & Pevzner, P. A. (2020). Metaviral SPAdes: assembly of viruses from metagenomic data. Bioinformatics, 36(14), 4126–4129. https://doi.org/10.1093/bioinformatics/btaa490
27. Kieft, K., Zhou, Z., & Anantharaman, K. (2020). VIBRANT: automated recovery, annotation and curation of microbial viruses, and evaluation of viral community function from genomic sequences. Microbiome, 8(1). https://doi.org/10.1186/s40168-020-00867-0
28. Roux, S., Enault, F., Hurwitz, B., & Sullivan, M. B. (2015). VirSorter: mining viral signal from microbial genomic data. PeerJ, 3, e985. https://doi.org/10.7717/peerj.985
29. Ren, J., Ahlgren, N. A., Lu, Y., Fuhrman, J. A., & Sun, F. (2017). VirFinder: a novel k-mer based tool for identifying viral sequences from assembled metagenomic data. Microbiome, 5(1). https://doi.org/10.1186/s40168-017-0283-5
30. Moraru, C. (2023). VirClust—A tool for hierarchical clustering, core protein detection and annotation of (Prokaryotic) viruses. Viruses, 15(4), 1007. https://doi.org/10.3390/v15041007
31. Nayfach, S., Camargo, A. P., Schulz, F., Eloe-Fadrosh, E. A., Roux, S., & Kyrpides, N. C. (2020). CheckV assesses the quality and completeness of metagenome-assembled viral genomes. Nature Biotechnology, 39(5), 578–585. https://doi.org/10.1038/s41587-020-00774-7
32. Schwengers, O., Jelonek, L., Dieckmann, M. A., Beyvers, S., Blom, J., & Goesmann, A. (2021). Bakta: rapid and standardized annotation of bacterial genomes via alignment-free sequence identification. Microbial Genomics, 7(11). https://doi.org/10.1099/mgen.0.000685
33. Hauser, M., Steinegger, M., & Söding, J. (2016). MMseqs software suite for fast and deep clustering and searching of large protein sequence sets. Bioinformatics, 32(9), 1323–1330. https://doi.org/10.1093/bioinformatics/btw006
34. Buchfink, B., Xie, C., & Huson, D. H. (2014). Fast and sensitive protein alignment using DIAMOND. Nature Methods, 12(1), 59–60. https://doi.org/10.1038/nmeth.3176
35. R. R. Wick (2018). Rrwick/Porechop. https://github.com/rrwick/Porechop
36. De Coster, W., D’Hert, S., Schultz, D. T., Cruts, M., & Van Broeckhoven, C. (2018). NanoPack: visualizing and processing long-read sequencing data. Bioinformatics, 34(15), 2666–2669. https://doi.org/10.1093/bioinformatics/bty149

37. Li, H. (2018). Minimap2: pairwise alignment for nucleotide sequences. Bioinformatics, 34(18), 3094–3100. https://doi.org/10.1093/bioinformatics/bty191
38. Davis, N. M., Proctor, D. M., Holmes, S., Relman, D. A., & Callahan, B. J. (2018). Simple statistical identification and removal of contaminant sequences in marker-gene and metagenomics data. Microbiome, 6(1). https://doi.org/10.1186/s40168-018-0605-2
39. Martí, J. M. (2019). Recentrifuge: Robust comparative analysis and contamination removal for metagenomics. PLOS Computational Biology, 15(4), e1006967. https://doi.org/10.1371/journal.pcbi.1006967

The International MetaSUB Consortium Authors List

Natasha Abdullah, Marcos Abraao, Ait-hamlat Adel, Muhammad Afaq, Faisal S. Al-Quaddoomi, Ireen Alam, Gabriela E. Albuquerque, Alex Alexiev, Kalyn Ali, Lucia E. Alvarado-Arnez, Sarh Aly, Jennifer Amachee, Maria G. Amorim, Majelia Ampadu, Muhammad Al-Fath Amran, Nala An, Watson Andrew, Harilanto Andrianjakarivony, Michael Angelov, Verónica Antelo, Catharine Aquino, Álvaro Aranguren, Luiza F. Araujo, Hitler Francois Vasquez Arevalo, Jenny Arevalo, Carme Arnan, Lucia Elena Alvarado Arnez, Fernanda Arredondo, Matthew Arthur, Freddy Asenjo, Thomas Saw Aung, Juliette Auvinet, Nuria Aventin, Sadaf Ayaz, Silva Baburyan, Abd-Manaaf Bakere, Katrin Bakhl, Thais F. Bartelli, Erdenetsetseg Batdelger, François Baudon, Kevin Becher, Carla Bello, Médine Benchouaia, Hannah Benisty, Anne-Sophie Benoiston, Joseph Benson, Diego Benítez, Juliana Bernardes, Denis Bertrand, Silvia Beurmann, Malay Bhattacharyya, Tristan Bitard-Feildel, Lucie Bittner, Christina Black, Guillaume Blanc, Brittany Blyther, Toni Bode, Julia Boeri, Bazartseren Boldgiv, Kevin Bolzli, Alexia Bordigoni, Ciro Borrelli, Sílvia Borràs, Sonia Bouchard, Jean-Pierre Bouly, Alicia Boyd, Gabriela P. Branco, Alessandra Breschi, Björn Brindefalk, Christian Brion, Alan Briones, Paulina Buczansla, Catherine M. Burke, Aszia Burrell, Irvind Buttar, Jalia Bynoe, Sven Bönigk, Kari O. Bøifot, Hiram Caballero, Xiao Wen Cai, Dayana Calderon, Angela Cantillo, Miguel Carbajo, Alessandra Carbone, Anais Cardenas, Katerine Carrillo, Laurie Casalot, Sofia Castro, Ana V. Castro, Astred Castro, Ana Valeria B. Castro, Simone Cawthorne, Jonathan Cedillo, Daniela Centrón, Salama Chaker, Jasna Chalangal, Allison Chan, Anastasia I. Chasapi, Starr Chatziefthimiou, Sreya Ray Chaudhuri, Akash Keluth Chavan, Francisco Chavez, Gregory Chem, Xiaoqing Chen, Michelle Chen, Jenn-Wei Chen, Ariel Chernomoretz, Allaeddine Chettouh, Daisy Cheung, Diana Chicas, Shirley Chiu, Hira Choudhry, Carl Chrispin, Kianna Ciaramella, Erika Cifuentes, Jake Cohen, David A. Coil, Sylvie Collin, Colleen Conger, Romain Conte, Flavia Corsi, Cecilia N. Cossio, Ana F. Costa, Delisia Cuebas, Bruno D'Alessandro, Katherine E. Dahlhausen, Aaron E. Darling, Pujita Das, Lucinda B. Davenport, Laurent David, Natalie R. Davidson, Gargi Dayama, Stéphane Delmas, Chris K. Deng, Chloé Dequeker, Alexandre Desert, Monika Devi, Felipe S. Dezem, Clara N. Dias, Timothy Ryan Donahoe, Sonia Dorado, LaShonda Dorsey, Steven Du, Alexandra Dutan, Naya Eady, Jonathan A. Eisen, Miar Elaskandrany, Lennard Epping, Juan P. Escalera-Antezana, Cassie L. Ettinger, Iqra Faiz, Luice Fan, Nadine Farhat, Emile Faure, Fazlina Fauzi, Charlie Feigin, Skye Felice, Laís Pereira Ferreira, Gabriel Figueroa, Aubin Fleiss, Denisse Flores, Jhovana L. Velasco Flores, Marcos A.S. Fonseca, Jonathan Foox, Juan Carlos Forero, Aaishah Francis, Kelly French, Pablo Fresia, Jacob Friedman, Alina Frolova, Jaime J. Fuentes, Josephine Galipon, Mathilde Garcia, Laura Garcia, Catalina García, Annie Geiger, Samuel M. Gerner, Sonia L. Ghose, Dao Phuong Giang, Matías Giménez, Donato Giovannelli, Dedan Githae, Spyridon

Gkotzis, Liliana Godoy, Samantha Goldman, Gaston H. Gonnet, Juana Gonzalez, Andrea Gonzalez, Camila Gonzalez-Poblete, Andrew Gray, Tranette Gregory, Charlotte Greselle, Sophie Guasco, Juan Guerra, Wolfgang Haehr, Sebastien Halary, Felix Hartkopf, Jaden J.A. Hastings, Arya Hawkins-Zafarnia, Nur Hazlin Hazrin-Chong, Eric Helfrich, Eva Hell, Tamera Henry, Samuel Hernandez, Pilar Lopez Hernandez, David Hess-Homeier, Lauren E. Hittle, Nghiem Xuan Hoan, Aliaksei Holik, Chiaki Homma, Irene Hoxie, Michael Huber, Elizabeth Humphries, Stephanie Hyland, Andrea Hässig, Roland Häusler, Nathalie Hüsser, Robert A. Petit III, Badamnyambuu Iderzorig, Mizuki Igarashi, Shaikh B. Iqbal, Shino Ishikawa, Sakura Ishizuka, Sharah Islam, Riham Islam, Kohei Ito, Sota Ito, Takayuki Ito, Tomislav Ivankovic, Tomoki Iwashiro, Sarah Jackson, JoAnn Jacobs, Marisano James, Soojin Jang, Marianne Jaubert, Marie-Laure Jerier, Esmeralda Jiminez, Ayantu Jinfessa, Ymke De Jong, Hyun Woo Joo, Guilllaume Jospin, Andre Kahles, Takema Kajita, Affifah Saadah Ahmad Kassim, Nao Kato, Amrit Kaur, Inderjit Kaur, Fernanda de Souza Gomes Kehdy, Vedbar S. Khadka, Shaira Khan, Mahshid Khavari, Michelle Ki, Gina Kim, Hyung Jun Kim, Sangwan Kim, Ryan J. King, Kaymisha Knights, Giuseppe KoLoMonaco, Ellen Koag, Nadezhda Kobko-Litskevitch, Michael Kozhar, Jonas Krebs, Nanami Kubota, Andrii Kuklin, Sheelta S. Kumar, Rachel Kwong, Lawrence Kwong, Ingrid Lafontaine, Juliana Lago, Tsoi Ying Lai, Elodie Laine, Manolo Laiola, Olha Lakhneko, Isha Lamba, Gerardo de Lamotte, Romain Lannes, Eleonora De Lazzari, Madeline Leahy, Hyunjung Lee, Yunmi Lee, Lucy Lee, Vincent Lemaire, Emily Leong, Marcus H.Y. Leung, Dagmara Lewandowska, Chenhao Li, Weijun Liang, Moses Lin, Priscilla Lisboa, Anna Litskevitch, Eric Minwei Liu, Tracy Liu, Mayra Arauco Livia, Yui Him Lo, Sonia Losim, Manon Loubens, Jennifer Lu, Alexander G. Lucaci, Simona Lysakova, Salah Mahmoud, Sara Abdul Majid, Denisse Maldonado, Krizzy Mallari, Tathiane M. Malta, Maliha Mamun, Dimitri Manoir, German Marchandon, Natalia Marciniak, Sonia Marinovic, Brunna Marques, Nicole Mathews, Yuri Matsuzaki, Vincent Matthys, Madelyn May, Elias McComb, Annabelle Meagher, Adiell Melamed, Wayne Menary, Katterinne N. Mendez, Ambar Mendez, Irène Mauricette Mendy, Irene Meng, Ajay Menon, Mark Menor, Roy Meoded, Nancy Merino, Cem Meydan, Karishma Miah, Mathilde Mignotte, Tanja Miketic, Wilson Miranda, Athena Mitsios, Ryusei Miura, Kunihiko Miyake, Maria D. Moccia, Natasha Mohan, Mohammed Mohsin, Karobi Moitra, Mauricio Moldes, Laura Molina, Jennifer Molinet, Orgil-Erdene Molomjamts, Eftar Moniruzzaman, Sookwon Moon, Isabelle de Oliveira Moraes, Mario Moreno, Maritza S. Mosella, Josef W. Moser, Christopher Mozsary, Amanda L. Muehlbauer, Oasima Muner, Muntaha Munia, Naimah Munim, Maureen Muscat, Tatjana Mustac, Cristina Muñoz, Francesca Nadalin, Areeg Naeem, Dorottya Nagy-Szakal, Mayuko Nakagawa, Ashanti Narce, Masaki Nasu, Irene González Navarrete, Hiba Naveed, Bryan Nazario, Narasimha Rao Nedunuri, Thomas Neff, Aida Nesimi, Wan Chiew Ng, Synti Ng, Gloria Nguyen, Elsy Ngwa, Agier Nicolas, Pierre Nicolas, Abdollahi Nika, Hosna Noorzi, Avigdor Nosrati, Houtan Noushmehr, Diana N. Nunes, Maria Obolenska, Kathryn O'Brien, Niamh B. O'Hara, Gabriella Oken, Rantimi A. Olawoyin, Javier Quilez Oliete, Kiara Olmeda, Tolulope Oluwadare, Itunu A. Oluwadare, Nils Ordioni, Jenessa Orpilla, Jacqueline Orrego, Melissa Ortega, Princess Osma, Israel O. Osuolale, Oluwatosin M. Osuolale, Mitsuki Ota, Francesco Oteri, Yuya Oto, Rachid Ounit, Christos A. Ouzounis, Subhamitra Pakrashi, Rachel Paras, Coral Pardo-Este, Young-Ja Park, Paulina Pastuszek, Suraj Patel, Jananan Pathmanathan, Andrea Patrignani, Manuel Perez, Ante Peros, Sabrina Persaud, Anisia Peters, Adam Phillips, María Piekar, Lisbeth Pineda, Melissa P. Pizzi, Alma Plaku, Alketa Plaku, Sofya Pozdniakova, Brianna Pompa-Hogan, María Gabriela Portilla, Leonardo Posada, Max Priestman, Bharath Prithiviraj, Sambhawa Priya, Phanthira Pugdeethosal, Catherine E. Pugh, Benjamin Pulatov, Angelika Pupiec, Tao Qing, María Paula Quiroga, Saher Rahiel, Savlatjon Rahmatulloev, Kannan Rajendran, Aneisa Ramcharan,

Adan Ramirez-Rojas, Shahryar Rana, Prashanthi Ratnanandan, Timothy D. Read, Hubert Rehrauer, Renee Richer, Alexis Rivera, Michelle Rivera, Alessandro Robertiello, Courtney Robinson, Paula Rodríguez, Xavier Rodó, Nayra Aguilar Rojas, Paul Roldán, Anyelic Rosario, Sandra Roth, Maria Ruiz, Stephen Eduard Boja Ruiz, Kaitlan Russell, Thais S. Sabedot, Mahfuza Sabina, Ikuto Saito, Yoshitaka Saito, Gustavo Adolfo Malca Salas, Cecilia Salazar, Kaung Myat San, Jorge Sanchez, Khaliun Sanchir, Ryan Sankar, Paulo Thiago de Souza Santos, Zulena Saravi, Kai Sasaki, Yuma Sato, Masaki Sato, Seisuke Sato, Ryo Sato, Kaisei Sato, Nowshin Sayara, Steffen Schaaf, Oli Schacher, Anna-Lena M. Schinke, Ralph Schlapbach, Christian Schori, Jason R. Schriml, Felipe Segato, Felipe Sepulveda, Marianna S. Serpa, Paola F. De Sessions, Juan C. Severyn, Heba Shaaban, Maheen Shakil, Sarah Shalaby, Aliyah Shari, Hyenah Shim, Hikaru Shirahata, Yuh Shiwa, Rania Siam, Ophélie Da Silva, Jordana M. Silva, Gwenola Simon, Shaleni K. Singh, Kasia Sluzek, Rebecca Smith, Eunice So, Núria Andreu Somavilla, Yuya Sonohara, Nuno Rufino de Sousa, Camila Souza, Jason Sperry, Nicolas Sprinsky, Stefan G. Stark, Antonietta La Storia, Kiyoshi Suganuma, Hamood Suliman, Jill Sullivan, Arif Asyraf Md Supie, Chisato Suzuki, Sora Takagi, Fumie Takahara, Naoya Takahashi, Kou Takahashi, Tomoki Takeda, Isabella K. Takenaka, Soma Tanaka, Anyi Tang, Yuk Man Tang, Emilio Tarcitano, Andrea Tassinari, Mahdi Taye, Alexis Terrero, Eunice Thambiraja, Antonin Thiébaut, Sade Thomas, Andrew M. Thomas, Yuto Togashi, Takumi Togashi, Anna Tomaselli, Masaru Tomita, Itsuki Tomita, Xinzhao Tong, Oliver Toth, Nora C. Toussaint, Jennifer M. Tran, Catalina Truong, Stefan I. Tsonev, Kazutoshi Tsuda, Takafumi Tsurumaki, Michelle Tuz, Yelyzaveta Tymoshenko, Carmen Urgiles, Mariko Usui, Sophie Vacant, Brandon Valentine, Laura E. Vann, Fabienne Velter, Valeria Ventorino, Patricia Vera-Wolf, Riccardo Vicedomini, Michael A. Suarez-Villamil, Sierra Vincent, Renee Vivancos-Koopman, Andrew Wan, Cindy Wang, Tomoro Warashina, Ayuki Watanabe, Samuel Weekes, Johannes Werner, David Westfall, Lothar H. Wieler, Michelle Williams, Silver A. Wolf, Brian Wong, Yan Ling Wong, Tyler Wong, Rasheena Wright, Tina Wunderlin, Ryota Yamanaka, Jingcheng Yang, Hirokazu Yano, George C. Yeh, Olena Yemets, Tetiana Yeskova, Shusei Yoshikawa, Laraib Zafar, Yang Zhang, Shu Zhang, Amy Zhang, Yuanting Zheng
