# Supplementary material for: A modular metagenomics analysis system for integrated multi-step data exploration
Source: bioRxiv. 2023 Apr 9:2023.04.09.536171. Preprint. [Version 1] doi: 10.1101/2023.04.09.536171 (PMC10104186; doi:10.1101/2023.04.09.536171)
Supplement: Supplementary file 1 [file media-1.pdf]

METHODS

CURRENTLY AVAILABLE MODULES

There five modules used in this proof-of-concept analysis of short-read metagenomics sequencing datasets to accomplish one of two purposes: i) MAG inference and quality-checking and/or ii) short-read taxonomic classification. The algorithms listed were chosen based on previously documented performance in benchmarking studies, as well as code stability and overall workflow compatibility. The input for each module is a `samples.csv` containing the absolute paths to the appropriate file formats.

MAG INFERENCE AND QUALITY-CHECKING

Module 1: Short-read Preprocessing

Raw sequencing datasets are filtered for low-quality bases, low-complexity regions in reads, and extremely short reads using fastp (1). Reads can optionally be deduplicated. Filtered reads are trimmed of adapters using Trimmomatic (2). If host read removal is selected, trimmed and filtered reads are mapped using Bowtie2 and Samtools with the 'very-sensitive' flag to the host reference genome (here, the human reference genome assembly GRCh38), and mapped reads removed (3,4). As a last-pass, BayesHammer is used to correct sequencing errors (5). FastQC and MultiQC are used to generate overviews (ex. parameters such as per-base quality scores, sequence duplication levels) of processed dataset quality (6,7).

Module 2: Short-read Assembly

The processed sequencing reads can be assembled using MetaSPAdes (with optional flags for metaviral and/or plasmid assembly also available), MegaHIT, or both (8,9). Here, only MetaSPAdes was used.

Module 3: MAG Binning

Processed sequencing reads are mapped back to the *de novo* assembled contigs using Bowtie2 and Samtools. This read coverage information, along with the contig sequences themselves, are used as input for the following binning algorithms: MetaBAT2, CONCOCT, VAMB, and MaxBin2 (10 – 13). The sets of MAGs inferred by each algorithm are used as input for DAS Tool, an ensemble binning algorithm, to generate a set of consensus MAGs scored based on the presence/absence of single-copy genes (SCGs) (14).

Module 4: MAG Quality-Checking

The consensus refined MAGs are quality-checked using an array of parameters. CheckM calculates completeness, which is based on the number of lineage-specific marker gene sets present in a MAG, and contamination, which is the number of over-represented multiple copies of a marker gene in a MAG (15). MAGs are classified using GTDB-Tk, which relies on approximately calculating average nucleotide identity

(ANI) to a database of reference genomes (16). For MAGs with a species classification, their contig content is compared to the species' reference genome and genome-based completion, misassembly, and non-alignment statistics calculated using QUAST (17).

Short-read Taxonomic Classification

Module 5: Short-read Taxonomic Classification

The processed sequencing reads can be classified using MetaPhlan4, Kraken2/Bracken, and XTree (18-21). All three tools were used here. To estimate the relative abundance of a taxon, MetaPhlan4 calculates marker gene coverage, Bracken calculates the proportion of reads assigned to a taxon with k-mer uniqueness-based scaling, and XTree estimates directly from unique k-mer proportions. Since each of these output reports are of different formats, the raw reports from each algorithm are standardized in format for easier comparisons downstream.

Results

Replicate	Step	Num. Reads	Prop. Raw Reads	Num. Bases	Prop. Init. Bases	Mean Read Len. (bp)
2-1	Raw dataset	96640632	1.000	14551786070	1.000	150.58
2-1	Low qual. removal	91458150	0.946	13236886105	0.910	144.73
2-1	Adapter removal	91458148	0.946	13236848517	0.910	144.73
2-1	Host read removal	91456270	0.946	13236583541	0.910	144.73
2-1	Error correct.	91301980	0.945	13213425350	0.908	144.72
2-1	Retrimming	91296746	0.945	12025434182	0.826	132
2-2	Raw dataset	16309506	1.000	2456000824	1.000	150.59
2-2	Low qual. removal	15473452	0.949	2212519190	0.901	142.99
2-2	Adapter removal	15473452	0.949	2212512184	0.901	142.99
2-2	Host read removal	15472112	0.949	2212323675	0.901	142.99
2-2	Error correct.	15370934	0.942	2197116065	0.895	142.94
2-2	Retrimming	15370002	0.942	1997060256	0.813	131
2-3	Raw dataset	205557564	1.000	30833634600	1.000	150
2-3	Low qual. removal	154341578	0.751	22770712368	0.739	147.53
2-3	Adapter removal	154341570	0.751	22770699193	0.739	147.53
2-3	Host read removal	154336372	0.751	22769988305	0.738	147.53
2-3	Error correct.	153290982	0.746	22613328878	0.733	147.52
2-3	Retrimming	153290962	0.746	21233391896	0.689	139

Supp. Table 1: Sample 2 sequencing dataset size reductions after each step of the short-read preprocessing module.

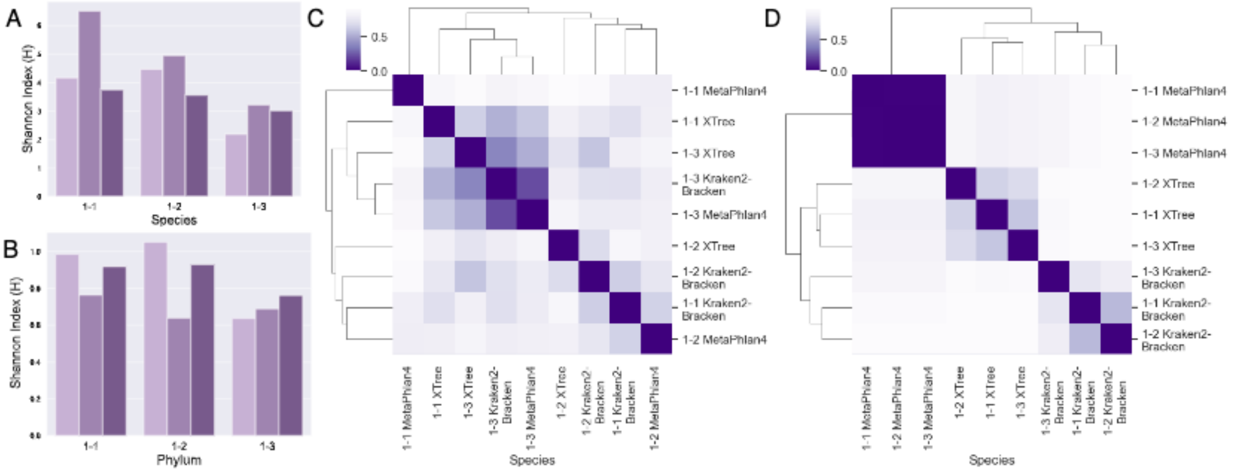

Supp. Figure 1: Alpha diversity as measured by the Shannon index (H) at the ranks of **(A)** species and **(B)** phylum. Beta diversity between the nine sets of species discovered in each of the three technical replicates by each of the three classifiers as measured by **(C)** Bray-Curtis dissimilarity and **(D)** Jaccard difference.

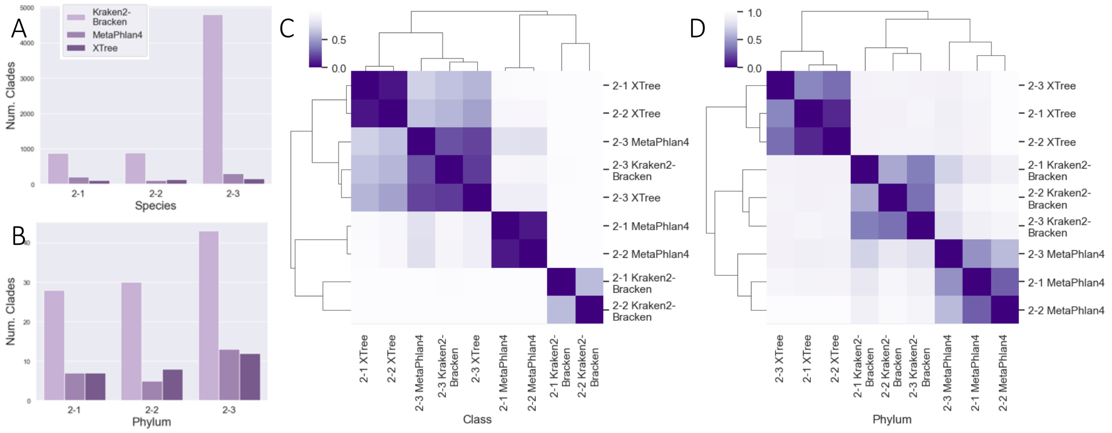

Supp. Figure 2: Sample 2 genetic diversity metrics. Alpha diversity as measured by the number of species at the ranks of **(A)** species and **(B)** phylum. Beta diversity between the nine sets of phyla discovered in each of the three technical replicates by each of the three classifiers as measured by **(C)** Bray-Curtis dissimilarity and **(D)** Jaccard difference.

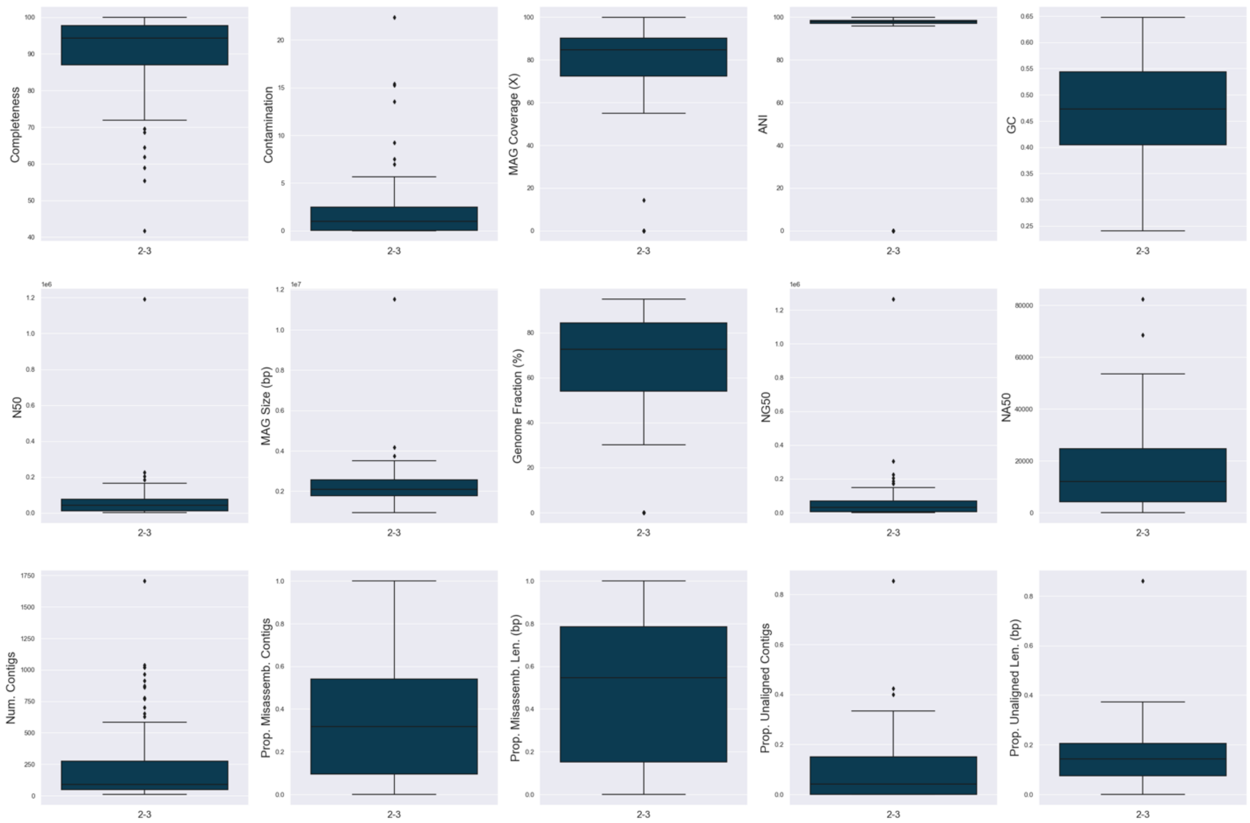

Supp. Figure 3: MAG quality-checking summary statistics for refined bins from the 2-3 dataset. From left to right then top to bottom: completeness, contamination, MAG coverage, ANI, GC (%), N50, MAG size, genome fraction (%), NG50, NA50, total number of contigs, proportion of contigs that are misassembled, proportion of sequence bases in misassembled contigs, proportion of contigs that do not align to the reference genome, proportion of sequence bases in unaligned contigs. The last 11 statistics were generated by QUAST.

References

1. Chen, S., Zhou, Y., Chen, Y., & Gu, J. (2018). Fastp: An ultra-fast all-in-one FASTQ preprocessor. Bioinformatics, 34(17), i884–i890.
2. Bolger, A., Lohse, M., & Usadel, B. (2014). Trimmomatic: A flexible trimmer for Illumina sequence data. Bioinformatics, 30(15), 2114–2120.
3. Langmead, B., & Salzberg, S. (2012). Fast gapped-read alignment with bowtie 2. Nature Methods, 9(4), 357–359.
4. Li, H., Handsaker, B., Wysoker, A., Fennell, T., Ruan, J., Homer, N., Marth, G., Abecasis, G., & Durbin, R. (2009). The sequence alignment/map format and SAMtools. Bioinformatics, 25(16), 2078–2079.
5. Nikolenko, S., Korobeynikov, A., & Alekseyev, M. (2013). Bayeshammer: Bayesian clustering for error correction in single-cell sequencing. BMC Genomics, 14(Suppl 1).
6. Andrews S. (2010) A quality control tool for high throughput sequence data. http://www.bioinformatics.babraham.ac.uk/projects/fastqc/.
7. Ewels, P., Magnusson, M., Lundin, S., & Käller, M. (2016). MultiQC: Summarize analysis results for multiple tools and samples in a single report. Bioinformatics, 32(19), 3047–3048.

8. Nurk, S., Meleshko, D., Korobeynikov, A., & Pevzner, P. (2017). Metaspades: A new versatile metagenomic assembler. Genome Research, 27(5), 824–834.
9. Li, D., Liu, C.M., Luo, R., Sadakane, K., & Lam, T.W. (2015). Megahit: An ultra-fast single-node solution for large and complex metagenomics assembly via succinct de Bruijn graph. Bioinformatics, 31(10), 1674–1676.
10. Kang, D., Li, F., Kirton, E., Thomas, A., Egan, R., An, H., & Wang, Z. (2019). MetaBAT 2: An adaptive binning algorithm for robust and efficient genome reconstruction from Metagenome Assemblies. PeerJ.
11. Alneberg, J., Bjarnason, B., Bruijn, I., Schirmer, M., Quick, J., Ijaz, U., Lahti, L., Loman, N., Andersson, A., Quince, C., & et al. (2014). Binning metagenomic contigs by coverage and composition. Nature Methods, 11(11), 1144–1146.
12. Nissen, J., Johansen, J., Allesøe, R., Sønderby, C., Armenteros, J., Grønbech, C., Jensen, L., Nielsen, H., Petersen, T., Winther, O., & et al. (2021). Improved metagenome binning and assembly using deep variational autoencoders. Nature Biotechnology, 39(5), 555–560.
13. Wu, Y.W., Simmons, B., & Singer, S. (2015). MaxBin 2.0: An automated binning algorithm to recover genomes from multiple metagenomic datasets. Bioinformatics, 32(4), 605–607.
14. Sieber, C., Probst, A., Sharrar, A., Thomas, B., Hess, M., Tringe, S., & Banfield, J. (2017). Recovery of genomes from metagenomes via a dereplication, aggregation, and scoring strategy. Nature Microbiology.
15. Parks, D., Imelfort, M., Skennerton, C., Hugenholtz, P., & Tyson, G. (2014). Checkm: Assessing the quality of microbial genomes recovered from isolates, single cells, and Metagenomes. Genome Research.
16. Chaumeil, P.A., Mussig, A., Hugenholtz, P., & Parks, D. (2019). GTDB-TK: A toolkit to classify genomes with the Genome Taxonomy Database. Bioinformatics.
17. Gurevich, A., Saveliev, V., Vyahhi, N., & Tesler, G. (2013). Quast: Quality Assessment Tool for Genome Assemblies. Bioinformatics, 29(8), 1072–1075.
18. Wood, D., Lu, J., & Langmead, B. (2019). Improved metagenomic analysis with Kraken 2. Genome Biology.
19. Lu, J., Breitwieser, F., Thielen, P., & Salzberg, S. (2017). Bracken: Estimating species abundance in metagenomics data. PeerJ Computer Science, 3.
20. Blanco-Miguez, A., Beghini, F., Cumbo, F., McIver, L., Thompson, K., Zolfo, M., Manghi, P., Dubois, L., Huang, K., Thomas, A., & et al. (2022). Extending and improving metagenomic taxonomic profiling with uncharacterized species with metaphlan 4. bioarXiv.
21. GabeAl. (2022). Gabeal/Utree: K-Mer searching with trees.
